# Direct Conversion of Hydrazones to Amines using Transaminases

**DOI:** 10.1002/cctc.202101008

**Published:** 2021-09-17

**Authors:** Eve M. Carter, Fabiana Subrizi, John M. Ward, Tom D. Sheppard, Helen C. Hailes

**Affiliations:** ^1^ Department of Chemistry University College London 20 Gordon Street London WC1H 0AJ UK; ^2^ Department of Biochemical Engineering University College London Gower Street, Bernard Katz Building London WC1E 6BT UK

**Keywords:** amines, biocatalysis, enzymes, hydrazones, transaminases

## Abstract

Transaminase enzymes (TAms) have been widely used for the amination of aldehydes and ketones, often resulting in optically pure products. In this work, transaminases were directly reacted with hydrazones in a novel approach to form amine products. Several substrates were investigated, including those with furan and phenyl moieties. It was determined that the amine yields increased when an additional electrophile was added to the reaction mixture, suggesting that they can sequester the hydrazine released in the reaction. Pyridoxal 5’‐phosphate (PLP), a cofactor for transaminases, and polyethylene glycol (PEG)‐aldehydes were both found to increase the yield of amine formed. Notably, the amination of (*S*)‐(−)‐1‐amino‐2‐(methoxymethyl)pyrrolidine (SAMP) hydrazones gave promising results as a method to form chiral β‐substituted amines in good yield.

## Graphical abstract

The reaction of aldehydes and ketones with transaminases has been widely used for producing amines. Here, a new transformation between hydrazones and transaminases was explored. Several substrates were investigated, forming a variety of amines in good yields. Amine yields were found to improve by the addition of electrophiles, thought to ‘trap’ the hydrazine released in the reaction.

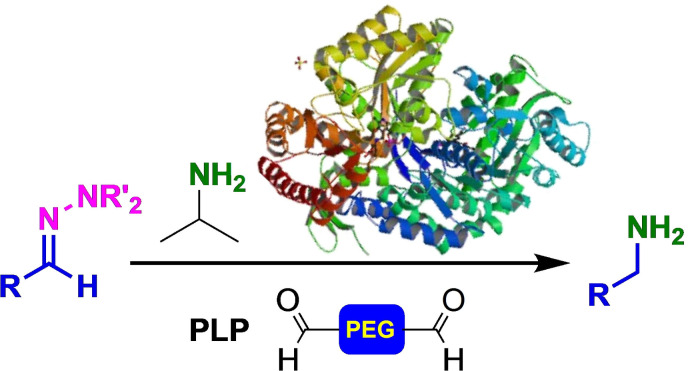

Transaminases (TAms), also known as aminotransferases, are typically used to reversibly transform a ketone or aldehyde group into an amine moiety using an amine donor and pyridoxal 5’‐phosphate (PLP) as the cofactor. When using prochiral ketones, the products can be single enantiomers and it is possible to access either enantiomer by switching between (*S*)‐ or (*R*)‐selective TAms.[^1^, ^2^] Chiral amines are very commonly found in pharmaceuticals and agrochemicals; therefore, the selective installation of a chiral amine is a desirable reaction. Indeed, transaminases have been used for numerous industrial applications, allowing for the stereoselective functionalisation of complex molecules.[^1^, ^2^, ^3^, ^4^, ^5^, ^6^, ^7^]

With the desire to improve the sustainability of synthetic transformations, there has been interest in extending the use of enzymes into new applications. Enzymes have many advantages when applied in synthetic processes compared to traditional organic synthetic methods, such as the use of milder reaction conditions and providing a more sustainable approach.[^5^, ^6^, ^7^] For example, there has recently been interest in reducing oximes using ene‐reductases to form chiral amines.^[8]^ Herein, we report the direct conversion of hydrazones to amines using transaminases, a transformation that has not previously been reported (Scheme 1).

**Scheme 1:**
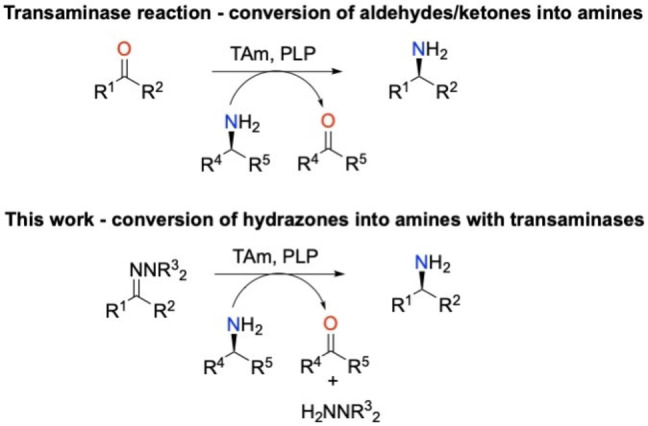
The amination of aldehydes and ketones using transaminases.[^1^, ^2^] This work investigates the reaction of transaminases with hydrazones to form amines.

Hydrazones are useful functional groups for a range of synthetic applications. The acidity of hydrogens at the α‐carbon of the hydrazone group, with a pKa of approximately 30, is far lower than in the parent carbonyl with a pKa of approximately 20, so conjugate bases are more reactive towards a number of electrophiles.^[9]^ The acidity at this α‐position is low enough to prevent the racemisation of chiral hydrazones, unlike the case with analogous carbonyls, so hydrazones are commonly used in asymmetric synthesis.^[10]^ Hydrazones also confer ‘Umpolung’ reactivity on the carbonyl unit which has been harnessed to control the reactivity of nearby functional groups including the cyclisation of furans to yield functionalised aromatics,^[11]^ and the regioselective cyclisation of aldoses into functionalised chiral tetrahydrofurans without the use of protecting groups.[^12^, ^13^] Given the wide utility of hydrazones in synthetic chemistry, we considered that the direct conversion of a hydrazone into an amine under mild conditions using a transaminase would be a useful reaction.

To probe whether this transformation might be achieved, an established colorimetric assay for screening TAms was performed. This colorimetric assay is based on the consumption of the amine donor 2‐(4‐nitrophenyl)ethan‐1‐amine **1**: if converted into the corresponding aldehyde **2**, a red precipitate **3** is generated (Scheme 2A).^[14]^ Hydrazones **4**–**9** were selected, synthesised and screened against multiple transaminases, together with benzaldehyde **10** as a positive control. Additional negative control reactions in the absence of enzyme were also performed. Seven TAms were chosen from our UCL TAm library based on their broad acceptance of substrates and tolerance towards organic solvents; *Chromobacterium violaceum* (*Cv‐*TAm),^[15]^ *Rhodobacter sphaeroides* (*Rh‐*TAm),^[16]^ *Mycobacterium vanbaalenii* (*Mv*‐TAm),^[17]^ *Arthrobacter sp*. variant ArRMut11 (*As‐*TAm)^[5]^ and three enzymes obtained from a functional metagenomics study on a domestic drain sample, and expressed from pQR2189, pQR2191 and pQR2208.^[18]^ Interestingly, the assay revealed that some hydrazones appeared to be transformed by the transaminases, with the aldehyde‐derived hydrazones **4**–**8** more readily accepted than the ketone‐derived hydrazone **9** (Scheme 2). In addition, the colorimetric assay with the corresponding aldehydes and ketone showed much higher activity towards the transaminases than with the hydrazones (see SI).[^3^, ^15^, ^16^, ^17^, ^18^]

**Scheme 2:**
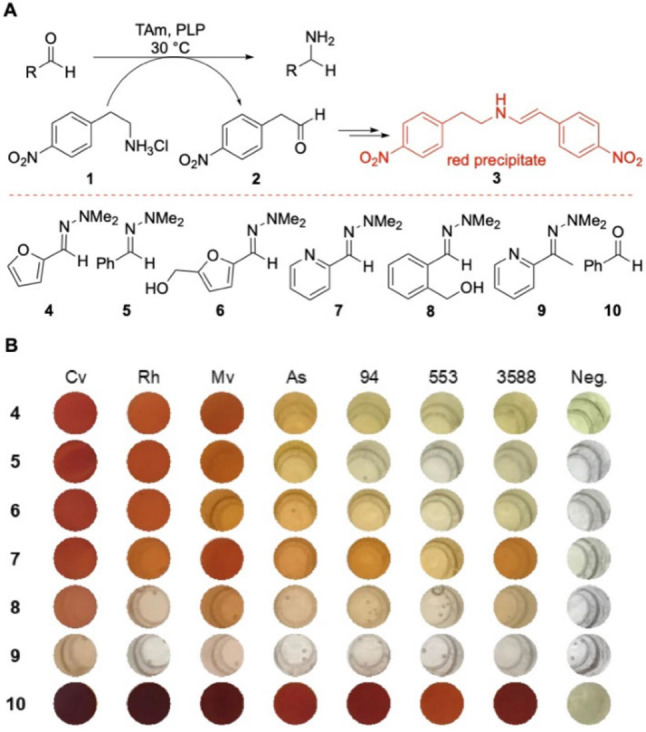
(**A**) Colorimetric assay using 2‐(4‐nitrophenyl)ethan‐1‐amine **1** as the amine donor which is converted into **3**.^[14]^ Hydrazone substrates used in the colorimetric assay. (**B**) Colorimetric assay of six hydrazones with seven enzymes: *Chromobacterium violaceum* (*Cv‐*TAm),^[15]^ *Rhodobacter sphaeroides* (*Rh‐*TAm),^[16]^ *Mycobacterium vanbaalenii* (*Mv*‐TAm),^[17]^ *Arthrobacter sp*. variant ArRMut11 (*As‐*TAm)^[5]^ and three metagenomic enzymes from a domestic drain: 94‐TAm pQR2189, 553‐TAm pQR2191 and 3588‐TAm pQR2208.^[18]^ *Reaction conditions*: total volume 200 μL containing amine **1** (25 mM), amine acceptor (10 mM), PLP (0.2 mM) and potassium phosphate buffer (100 mM, pH 8.0). A positive control was performed with benzaldehyde **10** as the amine acceptor. A negative control (−) was performed without any enzyme.

This novel transformation was then studied in more detail using substrate **4**, which was readily accepted by several TAms. Using the amine donors α‐methylbenzylamine (α‐MBA) and isopropylamine (IPA), the formation of the corresponding amine furfurylamine **11** was confirmed by HPLC against standards.^[19]^ It was also noted that higher yields were obtained at 45 °C compared to 37 °C. *Cv*‐TAm^[15]^ and IPA gave the best yields (∼20 %).

It was considered that the reaction may occur via hydrolysis of the hydrazone **4** *in situ*, potentially non‐enzymatically, forming the corresponding aldehyde, which is then directly aminated by the TAm to give amine **11** (Scheme 3A). Data to support this hypothesis was seen in negative control reactions; when no enzyme was present, under the same reaction conditions as for the TAm reaction, some aldehyde formation was observed by HPLC (see SI). It is often very difficult to completely hydrolyse a hydrazone, requiring harsh conditions, and so it was considered that this would be a useful transformation to explore further. It was also postulated that the hydrazine released during the reaction could inhibit the transaminase, so addition of an electrophile (E^+^) may help to drive the equilibrium towards the product by trapping hydrazine, improving the utility of the reaction. Care would have to be taken when considering suitable electrophiles, as other aldehydes may react with TAms to produce side products.

**Scheme 3:**
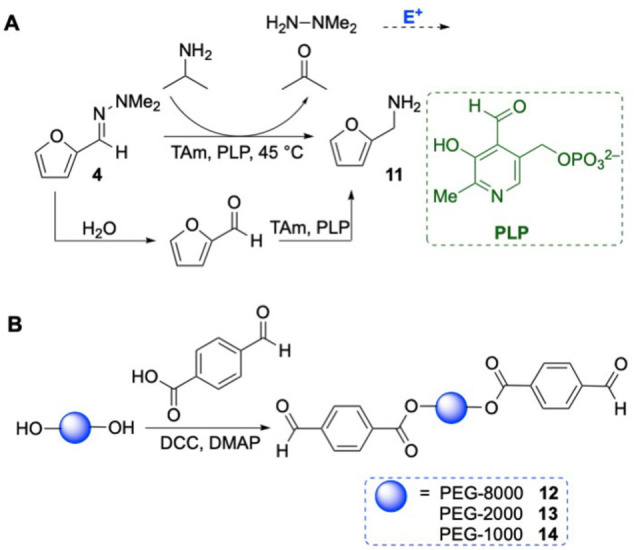
(**A**) Proposed route for the amination of hydrazones with transaminases. Reaction of the hydrazine released with an electrophile (E^+^) could be used to drive the reaction to amine product. (**B**) Preparation of PEG‐aldehydes **12**–**14** with average molecular weights of 8000, 2000 and 1000.

A commercially available polystyrene resin with a pendant benzaldehyde group was initially identified as a suitable electrophile as it would not fit into the active site of the TAm. However, no improvements were observed for the conversion of hydrazone **4** to amine **11** when the resin was added to the reaction. This was attributed to the poor swelling properties of polystyrene resins in aqueous conditions.^[20]^ Polyethylene glycol (PEG)‐supported aldehydes were then explored as PEG is well known to swell in water.^[21]^ A coupling reaction between 4‐formylbenzoic acid and different PEGs with average molecular weights of 8000, 2000 and 1000 was carried out and the products (**12**, **13** and **14**) used (Scheme 3B). Another suitable electrophile was thought to be the cofactor PLP, which could react with the hydrazine, and it is known not to negatively affect the activity of TAms.[^1^, ^2^]

Increases in yield of the amine **11** were observed with the PEG‐1000 **14** and PEG‐2000 **13** aldehydes, whereas no yield improvement was noted with the PEG‐8000 aldehyde **12**. This was thought to be because the higher molecular weight PEG‐aldehyde has poor solubility in water. Although the PEG‐aldehydes could react (reversibly) with the amine products (see SI), they nevertheless led to increased reaction yields and were therefore used in subsequent experiments. Yields of amine **11** were also increased when the concentration of PLP was increased, up to 70 % with 10 mM PLP and 10 mM or 15 mM of PEG‐1000 aldehyde **14** (Figure 1).

**Figure 1:**
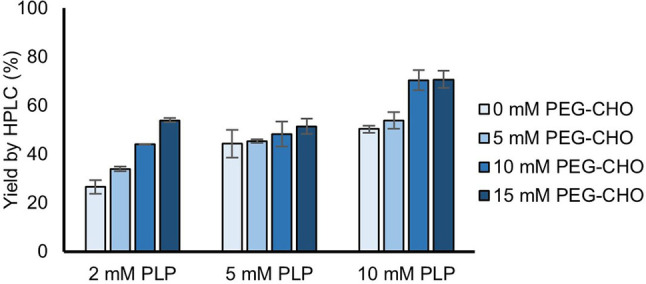
Yields of amine **11** when furfuryl hydrazone **4** (10 mM) was reacted with varying concentrations of PLP (2–10 mM) and PEG‐1000 aldehyde **14** (0–15 mM), IPA (500 mM), potassium phosphate buffer (pH 8.0, 100 mM) and *Cv*‐TAm crude cell lysate (50 μL) at 45 °C and 400 rpm for 24 h. Reactions were performed in triplicate and yields were determined by HPLC against product standards.

With these significant improvements in reaction yields with hydrazone **4**, alternative hydrazones were investigated. The dimethylhydrazone derivatives of several aldehydes linked to an aromatic ring (**5**, **15** and **16**) were explored. The yield was again found to increase as the concentration of PLP and PEG‐aldehyde were increased, up to 45 % for **17**, 67 % for **18** and 73 % for **19**. Notably the PEG‐aldehyde **13** was used routinely for wider applications, rather than **14** as, being a solid, it was easier to remove from the reactions. Higher yields were observed as the carbon chain increased in length, consistent with the increasing electrophilicity of the hydrazones (Scheme 4). Two of these reactions were performed on an enzyme preparative scale (25–30 mL, 10 mM) with yields of 38 % for **18** and 59 % for **19** by analytical HPLC. As an example, amine **19** was isolated, giving a 52 % yield.

**Scheme 4:**
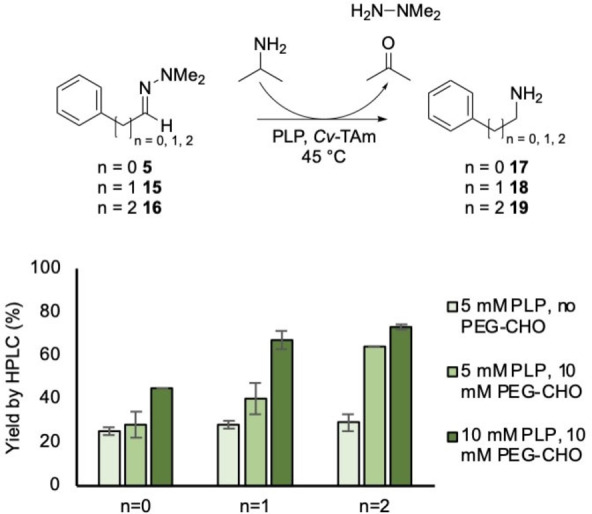
Reactions of phenyl hydrazones **5**, **15**, **16** (10 mM) to give amines **17**, **18**, **19** that were reacted with varying concentrations of PLP (5–10 mM) and PEG‐2000 aldehyde **13** (0–10 mM), IPA (500 mM), potassium phosphate buffer (pH 8.0, 100 mM) and *Cv*‐TAm crude cell lysate (50 μL) at 45 °C and 400 rpm for 24 h. Reactions were performed in triplicate and yields were determined by HPLC against product standards.

To demonstrate the potential utility of the reaction, a hydrazone that is used extensively as a chiral auxiliary was explored. (*S*)‐(−)‐1‐Amino‐2‐(methoxymethyl)pyrrolidine (SAMP, **20**) and the opposite enantiomer, (*R*)‐1‐amino‐2‐(methoxymethyl)pyrrolidine (RAMP) have been used for a number of applications in asymmetric synthesis including the asymmetric α‐alkylation of aldehydes and ketones.[^22^, ^23^] Hydrazone **21** was prepared via the asymmetric alkylation of hydrazone **22** derived from hydrocinnamaldehyde (Scheme 5). Direct hydrolysis to remove the hydrazone auxiliary from compounds such as **21** is typically unsuccessful, and destructive methods are often required which cleave the N−N bond via reduction with catecholborane then hydrogenation with Raney nickel to form the corresponding amine **23** ^[24]^ (Scheme 5). To investigate this reaction under much milder conditions using TAms, compounds **21** and **22** were screened against *Cv*‐TAm in the presence of PLP and the PEG‐2000 aldehyde **13**. Pleasingly, the SAMP group was removed in a good yield from both **21** and **22** and they were converted into the corresponding amines **23** and **19** in one step with yields of over 70 % (Scheme 5). The enantiomeric excess (*ee*) of the amine product **23** was determined by chiral HPLC as 90 %. It was also notable here that addition of the PEG‐aldehyde **13** significantly enhanced yields by removing any hydrazine that could detrimentally affect the enzyme. As well as being a higher yielding procedure than reported in the literature to convert these compounds into amines (∼50 %), this novel method avoids the use of toxic/hazardous reagents. Additionally, it would be possible to recover the SAMP **20** from the reaction. This reaction was performed on an enzyme preparative scale (25 mL, 10 mM) with a yield of 64 % by analytical HPLC and amine **23** was isolated in a 48 % yield.

**Scheme 5:**
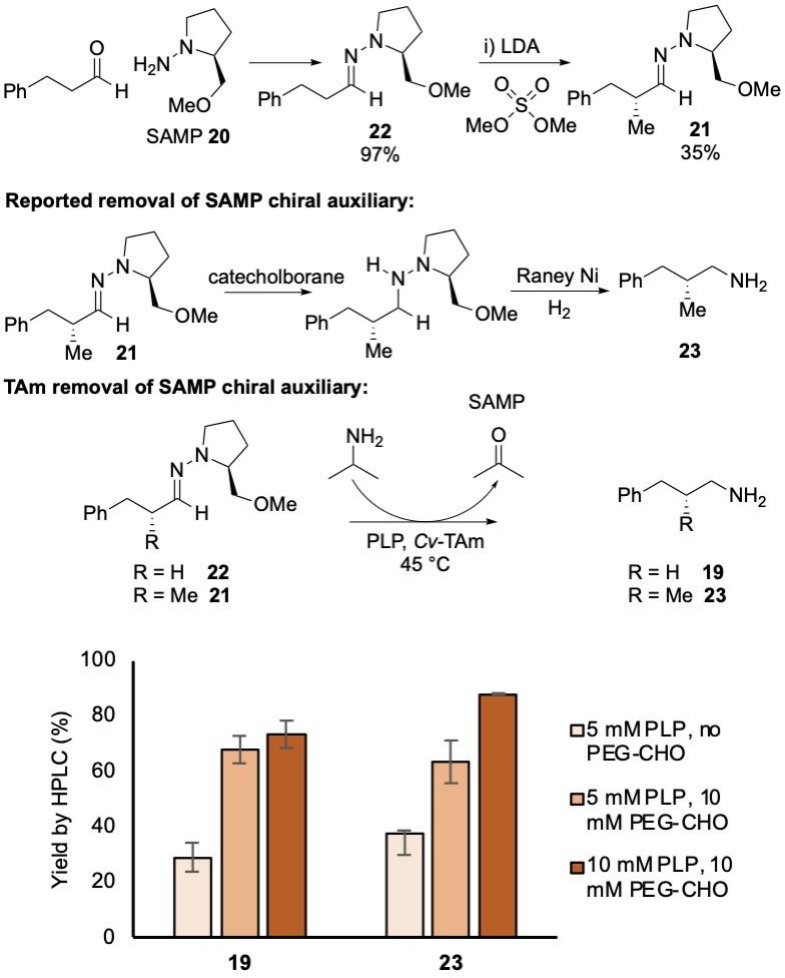
The synthesis of **22** and **21**. The SAMP hydrazone **21** is traditionally converted to the chiral amine **23** using catecholborane then Raney nickel.^[22]^ Here, a transaminase was used to remove the SAMP chiral auxiliary, forming amines **19** and **23** in one step: SAMP hydrazones **22** and **21** (10 mM) were reacted with varying concentrations of PLP (5‐10 mM) and PEG‐2000 aldehyde **13** (0–10 mM), IPA (500 mM), potassium phosphate buffer (pH 8.0, 100 mM) and *Cv*‐TAm crude cell lysate (50 μL) at 45 °C and 400 rpm for 24 h. Reactions were performed in duplicate and yields were determined by HPLC against product standards.

In summary, here we report the novel reaction of transaminases with hydrazones to directly generate amines. It was found that ‘trapping’ the hydrazine released in the transformation using PLP and PEG‐supported aldehydes considerably increased yields in many cases. Mechanistically, the reaction is believed to proceed via hydrolysis of the hydrazone, forming the aldehyde *in situ*, which then reacts with the TAm to form the corresponding amine. This provides an effective hydrolysis of the hydrazone under mild conditions, as the enzyme continually removes the free aldehyde from solution by converting it into the corresponding amine. The applicability of the reaction was demonstrated using SAMP hydrazones, providing a high yielding and sustainable method to form β‐substituted chiral amines. This is a useful reaction to add to the growing repertoire of enzymatic transformations.

## Conflict of interest

The authors declare no conflict of interest.

## Supporting information

As a service to our authors and readers, this journal provides supporting information supplied by the authors. Such materials are peer reviewed and may be re‐organized for online delivery, but are not copy‐edited or typeset. Technical support issues arising from supporting information (other than missing files) should be addressed to the authors.

Supporting Information
