# Supplementary material for: Direct Conversion of Hydrazones to Amines using Transaminases
Source: ChemCatChem. 2021 Sep 17;13(21):4520–3. doi: 10.1002/cctc.202101008 (PMC9292518; doi:10.1002/cctc.202101008)
Supplement: Supplementary file 1 — Supporting Information [file CCTC-13-4520-s001.pdf]

ChemCatChem

Supporting Information

Direct Conversion of Hydrazones to Amines using Transaminases

Eve M. Carter, Fabiana Subrizi, John M. Ward, Tom D. Sheppard, and Helen C. Hailes*

Author Contributions

H.H. Conceptualization:Equal; Methodology:Supporting; Supervision:Lead; Writing – original draft:Equal; Writing – review & editing:Lead
E.C. Conceptualization:Supporting; Investigation:Lead; Methodology:Lead; Writing – original draft:Equal
F.S. Conceptualization:Equal; Investigation:Supporting; Writing – review & editing:Supporting
J.W. Conceptualization:Supporting; Methodology:Supporting; Writing – review & editing:Supporting
T.S. Conceptualization:Supporting; Methodology:Supporting; Supervision:Supporting; Writing – review & editing:Supporting

Table of Contents

1 Synthetic Methods 1

1.1 General methods 1

1.2 Synthesis of hydrazones 2

1.3 Synthesis of racemic 2-methyl-3-phenylpropan-1-amine 7

1.4 Synthesis of PEGs with pendant aldehydes 8

1.5 Control reactions 10

2 Chemical Biology 12

2.1 General methods 12

2.2 Protein expression 12

2.3 Assays 13

3 Analytical HPLC 17

4 NMR spectra 21

5 References 36

1 Synthetic Methods

1.1 General methods

All reagents were obtained from commercial suppliers and used without further purification. Solvents were evaporated under reduced pressure using a Büchi rotary evaporator. Room temperature is defined as between 18-22 °C. Flash column chromatography was carried out using Geduran Silicagel 60, particle size 40-63 µm. All chemical reactions were followed by thin layer chromatography (TLC) when practical, using Merck aluminium backed Silicagel 60 F_{254} fluorescent treated silica which was visualised under UV light (254 nm or 365 nm). In addition, TLC plates were stained with aqueous basic potassium permanganate solution.

^{1}H and ^{13}C NMR spectra were recorded using Bruker Avance Neo 700 and Bruker Avance III 600. Chemical shifts (δ) are quoted in parts per million (ppm) and are referenced to the residual solvent peak: $CDCl_3$ (δ 7.26 ppm in ^{1}H and δ 77.2 ppm in ^{13}C). Coupling constants (*J*) are quoted in Hertz (Hz). The ^{1}H NMR spectra are reported as follows: ppm (multiplicity, coupling constants, number of protons, assignment). NMR assignments use numbering independent from IUPAC, using two-dimensional (COSY, HSQC, HMBC) NMR spectroscopy to assist the assignment.

Infrared (IR) spectra were recorded on Perkin Elmer Spectrum 100 FTIR Spectrometer operating in ATR mode. Absorption maxima (ν_{max}) are reported in wavenumbers (cm^{-1}). Only selected absorption maxima are reported.

Low Resolution Mass Spectra (LRMS) and **High Resolution Mass Spectra (HRMS)** were recorded on a Waters LCT Premier Q-TOF or Waters Aquity UPLC-SQD operating in ESI mode, or Thermo MAT900 if EI/CI, or on a Micromass MALDI-TOF depending on the compound.

Melting points (Mp) were recorded on a Stuart SMP11 machine in degrees Celsius (°C).

Optical rotations ($[\alpha]_D$) were recorded on a Bellingham + Stanley ADP430 polarimeter.

1.2 Synthesis of hydrazones

2-(Furan-2-ylmethylene)-1,1-dimethylhydrazine 4[1]

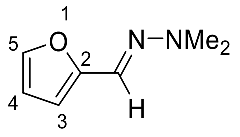

To a solution of furfural (1.00 mL, 12.1 mmol) in water (200 mL) was added *N*,*N*-dimethylhydrazine (1.19 mL, 15.7 mmol) and the resulting mixture stirred for 4 h. The solution was extracted with EtOAc (3 × 200 mL). The combined organic layers were dried with $MgSO_4$, filtered, and concentrated under reduced pressure. The crude product was purified by column chromatography (1:19, EtOAc:*n*-hexane) to yield the product as an orange oil (1.07 g, 64%). **^{1}H NMR** (600 MHz; $CDCl_3$) δ 7.35 (d, *J* = 1.8 Hz, 1H, 5-H), 7.10 (s, 1H, N=CH), 6.37 (dd, *J* = 3.4, 1.8 Hz, 1H, 4-H), 6.34 (d, *J* = 3.4 Hz, 1H, 3-H), 2.93 (s, 6H, NMe_2); **^{13}C NMR** (151 MHz; $CDCl_3$) δ 152.2, 142.1, 123.5, 111.4, 107.4, 43.0; ***m/z*** [HRMS ESI+] found $[M+H]^+$ 139.0867. $C_7H_{11}N_2O$ requires 139.0871. All spectral data corresponds to those given in the literature.[1]

2-Benzylidene-1,1-dimethylhydrazine 5[2]

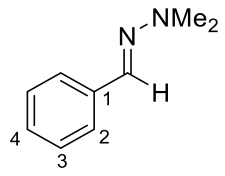

To a solution of benzaldehyde (0.45 mL, 4.4 mmol) in anhydrous ethanol (40 mL) under argon was added *N*,*N*-dimethylhydrazine (0.50 mL, 6.6 mmol) and the resulting mixture stirred at 50 °C for 3 h. The solution was diluted with water (50 mL) and extracted with dichloromethane (3 × 50 mL). The combined organic layers were dried with $MgSO_4$, filtered, and concentrated under reduced pressure to yield the product as a yellow oil (490 mg, 75%). **^{1}H NMR** (600 MHz; $CDCl_3$) δ 7.63 – 7.57 (m, 2H, 2-H), 7.36 – 7.32 (m, 2H, 3-H), 7.27 (s, 1H, N=CH), 7.26 – 7.22 (m, 1H, 4-H), 2.98 (s, 6H, NMe_2); **^{13}C NMR** (151 MHz; $CDCl_3$) δ 137.0, 133.0, 128.6, 127.4, 125.7, 42.9; ***m/z*** [HRMS ESI+] found $[M+H]^+$ 149.1076. $C_9H_{13}N_2$ requires 149.1079. All spectral data corresponds to those given in the literature.[2]

(5-((2,2-Dimethylhydrazineylidene)methyl)furan-2-yl)methanol 6[3]

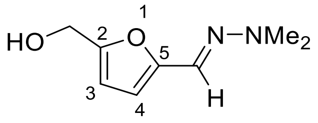

To a solution of hydroxymethylfurfural (630 mg, 5.0 mmol) in water (30 mL) was added *N*,*N*-dimethylhydrazine (0.49 mL, 6.5 mmol) and the resulting mixture was stirred at 50 °C for 2 h. The solution was extracted with EtOAc (3 × 30 mL). The combined organic layers were dried with $MgSO_4$, filtered, and concentrated under reduced pressure to yield the product as an orange oil (840 mg, 100%). **^{1}H NMR** (600 MHz; $CDCl_3$) δ 7.07 (s, 1H, N=CH), 6.30 (d, *J* = 3.3 Hz, 1H, 4-H), 6.28 (d, *J* = 3.3 Hz, 1H, 3-H), 4.60 (s, 2H, CH_2), 2.93 (s, 6H, NMe_2); **^{13}C NMR** (151 MHz; $CDCl_3$) δ 153.8, 152.1, 123.5, 109.6, 108.2, 57.6, 42.9; ***m/z*** [HRMS ESI+] found $[M+H]^+$ 169.0973. $C_8H_{13}N_2O_2$ requires 169.0977. All spectral data corresponds to those given in the literature.[3]

2-((2,2-Dimethylhydrazineylidene)methyl)pyridine 7[4]

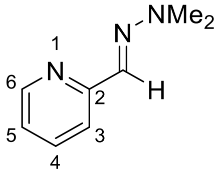

To a solution of 2-pyridinecarboxaldehyde (0.48 mL, 5.0 mmol) in anhydrous ethanol (40 mL) under argon was added *N*,*N*-dimethylhydrazine (0.57 mL, 7.5 mmol) and the resulting mixture was stirred at 50 °C for 3 h. The solution was diluted with water (50 mL) and extracted with EtOAc (3 × 50 mL). The combined organic layers were dried with $MgSO_4$, filtered, and concentrated under reduced pressure to yield the product as a yellow oil (680 mg, 91%). **^{1}H NMR** (700 MHz; $CDCl_3$) δ 8.40 (ddd, *J* = 4.9, 1.7, 1.0 Hz, 1H, 6-H), 7.71 – 6.67 (m, 1H, 3-H), 7.52 – 7.48 (m, 1H, 4-H), 7.23 – 7.17 (s, 1H, N=CH), 6.97 (ddd, *J* = 7.6, 4.9, 1.2 Hz, 1H, 5-H), 2.96 (s, 6H, NMe_2); **^{13}C NMR** (176 MHz; $CDCl_3$) δ 155.9, 148.9, 136.0, 131.5, 121.3, 118.7, 42.5; ***m/z*** [HRMS ESI+] found $[M+H]^+$ 150.1029. $C_8H_{11}N_3$ requires 150.1031. All spectral data corresponds to those given in the literature.[4]

1,3-Dihydroisobenzofuran-1-ol/2-(hydroxymethyl)benzaldehyde 8a

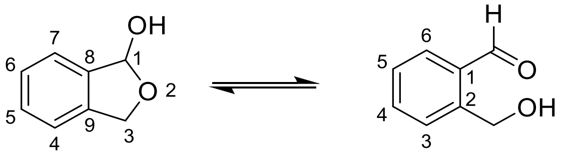

I II

To a solution of phthalide (450 mg, 3.3 mmol) in anhydrous dichloromethane (20 mL) under argon at -78 °C was added DIBAL-H (1 M in THF, 5.0 mL, 5.0 mmol) dropwise. The solution was stirred for 0.5 h before quenching with the dropwise addition of methanol (2 mL) at -78 °C and then addition of sat. Rochelle's salt (10 mL). The solution was diluted with water (40 mL) and then extracted with dichloromethane (3 × 40 mL). The combined organic layers were dried with $MgSO_4$, filtered, and concentrated under reduced pressure to yield the product as a sticky white solid (400 mg, 88%). **IR** (film) ν_{max}/cm^{-1}: 3356, 2921, 2869, 1688, 1573; **^{1}H NMR** (700 MHz; $CDCl_3$) **(I):** δ 7.43 (d, *J* = 7.4 Hz, 1H, 7-H), 7.37 (m, 1H, 5-H), 7.35 – 7.31 (m, 1H, 6-H), 7.25 (d, *J* = 7.4 Hz, 1H, 4-H), 6.47 (d, *J* = 6.1 Hz, 1H, 1-H), 5.21 (m, 1H, 3-$\underline{H}$H), 4.99 (d, *J* = 12.6 Hz, 1H, 3-H$\underline{H}$), 3.81 (d, *J* = 6.1 Hz, 1H, OH); **(II):** δ 10.07 (s, 1H, CHO), 7.85 (dd, *J* = 7.5, 1.4 Hz, 1H, 6-H), 7.60 (m, 1H, 4-H), 7.53 (m, 1H, 5-H), 7.49 (d, *J* = 7.5 Hz, 1H, 3-H), 4.82 (d, *J* = 7.0 Hz, 2H, CH_2), 3.72 (t, *J* = 7.0 Hz, 1H, OH); **^{13}C NMR** (176 MHz; $CDCl_3$) for (**I**) δ 142.6, 139.1, 129.4, 128.0, 123.1, 121.2, 101.9, 72.0; for (**II**) δ 195.2, 139.5, 135.8, 134.7, 134.3, 129.8, 128.4, 64.0; ***m/z*** [ESI+] 159 ([M+Na]$^+$, 100%); ***m/z*** [HRMS ESI+] found [M+H]$^+$ 137.0606. $C_8H_9O_2$ requires 137.0603.

2-((2,2-Dimethylhydrazineylidene)methyl)phenyl)methanol 8

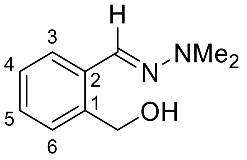

To a solution of **8a** (330 mg, 2.4 mmol) in anhydrous ethanol (20 mL) under argon was added *N,N*-dimethylhydrazine (0.55 mL, 7.3 mmol) and the resulting mixture was stirred at 50 °C for 3 h. The solution was diluted with water (50 mL) and extracted with EtOAc (3 × 50 mL). The combined organic layers were dried with $MgSO_4$, filtered, and concentrated under reduced pressure to yield the product as a yellow oil (370 mg, 86%). **IR** (film) ν_{max}/cm^{-1}: 3357, 2858, 1690; **^{1}H NMR** (700 MHz; $CDCl_3$) δ 7.41 – 7.35 (m, 2H, 5-H and N=CH), 7.33 – 7.29 (m, 2H, 4-H and 6-H), 7.24 – 7.21 (m, 1H, 3-H), 5.21 (br s, 1H, OH), 4.62 (s, 2H, CH_2), 3.00 (s, 6H, NMe_2); **^{13}C NMR** (176 MHz; $CDCl_3$) δ 138.0, 134.9, 134.9, 130.4, 130.0, 127.9, 127.7, 64.9, 42.8; ***m/z*** [ESI+] 161 ([M-OH]$^+$, 54%), 179 ([M+H]$^+$, 100%); ***m/z*** [HRMS ESI+] found [M+H]$^+$ 179.1176. $C_{10}H_{15}N_2O$ requires 179.1184.

2-(1-(2,2-Dimethylhydrazineylidene)ethyl)pyridine 9

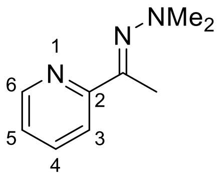

To a solution of 2-acetylpyridine (0.79 mL, 7.0 mmol, 1 equiv.) in anhydrous ethanol (50 mL) under argon was added *N,N*-dimethylhydrazine (1.6 mL, 21 mmol, 3 equiv.) and the resulting mixture was stirred at 50 °C for 5 h. The solution was diluted with water (200 mL) and extracted with EtOAc (3 × 150 mL). The combined organic layers were dried with $MgSO_4$, filtered, and concentrated under reduced pressure to yield the product as a yellow oil (320 mg, 28%). **IR** (film) ν_{max}/cm^{-1}: 2959, 1582; **^{1}H NMR** (700 MHz; $CDCl_3$) δ 8.53 (ddd, *J* = 4.8, 1.8, 0.9 Hz, 1H, 6-H), 7.98 (dd, *J* = 8.7, 0.9 Hz, 1H, 3-H), 7.60 (ddd, *J* = 8.7, 7.6, 1.8 Hz, 1H, 4-H), 7.18 (ddd, *J* = 7.6, 4.8, 1.2 Hz, 1H, 5-H), 2.62 (s, 3H, NMe), 2.61 (s, 3H, NMe), 2.38 (s, 3H, CH_3); **^{13}C NMR** (176 MHz; $CDCl_3$) δ 161.4, 156.7, 148.6, 136.1, 123.7, 120.8, 47.3, 14.8; ***m/z*** [ESI+] 164 ($[M+H]^+$, 100%); ***m/z*** [HRMS ESI+] found $[M+H]^+$ 164.1182. $C_9H_{14}N_3$ requires 164.1188.

1,1-Dimethyl-2-(2-phenylethylidene)hydrazine 15[5]

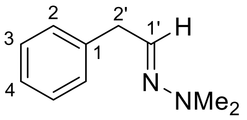

To a solution of phenylacetaldehyde (0.58 mL, 5.0 mmol) in anhydrous ethanol (40 mL) under argon was added *N,N*-dimethylhydrazine (0.76 mL, 10 mmol) and the resulting mixture was stirred for 5 h. The solution was diluted with water (100 mL) and extracted with EtOAc (3 × 50 mL). The combined organic layers were dried with $MgSO_4$, filtered, and concentrated under reduced pressure. The crude product was purified by column chromatography (1:20, EtOAc:*n*-hexane) to yield the product as a yellow oil (600 mg, 74%). **^{1}H NMR** (700 MHz; $CDCl_3$) δ 7.33 –7.31 (m, 2H, 3-H), 7.27 – 7.20 (m, 3H, 2-H and 4-H), 6.70 (t, *J* = 5.8 Hz, 1H, 1'-H), 3.59 (d, *J* = 5.8 Hz, 2H, 2'-H), 2.77 (s, 6H, NMe_2); **^{13}C NMR** (176 MHz; $CDCl_3$) δ 138.7, 137.2, 129.0, 128.7, 126.5, 43.4, 39.7; ***m/z*** [ESI+] 163 ($[M+H]^+$, 100%). All spectral data corresponds to those given in the literature.[5]

1,1-Dimethyl-2-(3-phenylpropylidene)hydrazine 16[6]

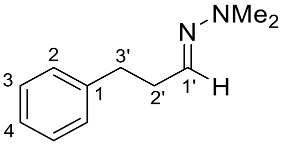

To a solution of hydrocinnamaldehyde (1.32 mL, 10.0 mmol) in anhydrous ethanol (50 mL) under argon was added *N,N*-dimethylhydrazine (1.52 mL, 20.0 mmol) and the resulting mixture was stirred for 18 h. The solution was diluted with water (200 mL) and extracted with EtOAc (3 × 150 mL). The combined organic layers were dried with $MgSO_4$, filtered, and concentrated under reduced pressure to yield the product as a yellow oil (1.66 g, 94%). **^{1}H NMR** (700 MHz; $CDCl_3$) δ 7.31 – 7.28 (m, 2H,

3-H), 7.24 – 7.17 (m, 3H, 2-H and 4-H), 6.67 (t, *J* = 5.4 Hz, 1H, 1’-H), 2.84 – 2.79 (m, 2H, 3’-H), 2.74 (s, 6H, NMe_2), 2.60 – 2.55 (m, 2H, 2’-H); **^{13}C NMR** (176 MHz; $CDCl_3$) δ 141.6, 138.1, 128.6, 128.5, 126.0, 43.4, 34.9, 34.2; ***m/z*** [ESI+] 177 ($[M+H]^+$, 100%), 218 ($[M+CH_3CN+H]^+$, 10%). All spectral data corresponds to those given in the literature.[6]

(2*S*,1*E*)-*N*-(2-(Methoxymethyl)pyrrolidin-1-yl)-3-phenylpropan-1-imine 22[7]

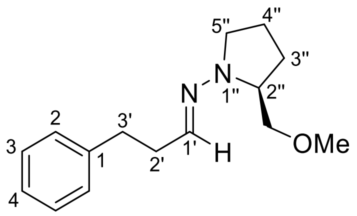

To a solution of (*S*)-(−)-1-amino-2-(methoxymethyl)pyrrolidine (SAMP) (0.50 mL, 3.7 mmol) in anhydrous dichloromethane (4 mL) under argon at 0 °C was added hydrocinnamaldehyde (0.49 mL, 3.7 mmol) and the reaction stirred at room temperature for 3 h. The solution was diluted with water (20 mL) and then extracted with dichloromethane (3 × 20 mL). The combined organic layers were dried with $MgSO_4$, filtered, and concentrated under reduced pressure to yield the product as a pale yellow oil (890 mg, 97%). $[\alpha]_D^{22}$ = -104 (*c* 0.5, diethyl ether); **^{1}H NMR** (700 MHz; $CDCl_3$) δ 7.30 – 7.27 (m, 2H, 3-H), 7.23 – 7.17 (m, 3H, 2-H and 4-H), 6.66 (t, *J* = 5.5 Hz, 1H, 1’-H), 3.58 – 3.53 (m, 1H, C<u>H</u>H-OMe), 3.44 – 3.40 (m, 2H, CH<u>H</u>-OMe and 2”-H), 3.38 (s, 3H, OMe), 3.36 – 3.32 (m, 1H, 5”-<u>H</u>H), 2.84 – 2.77 (m, 2H, 3’-H), 2.71 (m, 1H, 5”-H<u>H</u>), 2.59 – 2.51 (m, 2H, 2’-H), 2.04 – 1.85 (m, 3H, 3”-<u>H</u>H and 4”-H), 1.83 – 1.74 (m, 1H, 3”-H<u>H</u>); **^{13}C NMR** (176 MHz; $CDCl_3$) δ 141.8, 137.9, 128.6, 128.5, 126.0, 75.0, 63.6, 59.4, 50.6, 35.0, 34.3, 26.7, 22.4; ***m/z*** [ESI+] 247 ($[M+H]^+$, 100%), 288 ($[M+CH_3CN+H]^+$, 6%). All spectral data corresponds to those given in the literature.[7]

(2*R*,1*E*)-*N*-((2*S*)-2-(Methoxymethyl)pyrrolidin-1-yl)-2-methyl-3-phenylpropan-1-imine 21

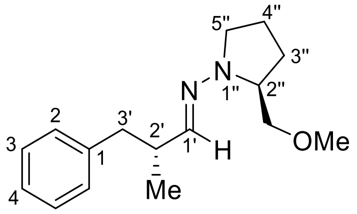

A solution of diisopropylamine (0.31 mL, 2.2 mmol) in anhydrous THF (5 mL) under argon was cooled to 0 °C and *n*-BuLi (2.5 M in hexanes, 0.98 mL) was added dropwise. The solution was stirred at 0 °C for 0.5 h then **22** (480 mg, 1.9 mmol) in anhydrous THF (1 mL) was added dropwise and the reaction was stirred for 3 h. The reaction was cooled to -78 °C then dimethyl sulfate (0.21 mL,

2.2 mmol) was added dropwise and the reaction stirred for 2 h before being quenched with sat. NH_4Cl and warmed to room temperature. The solution was diluted with water (20 mL) and extracted with diethyl ether (3 × 20 mL). The combined organic layers were dried with $MgSO_4$, filtered, and concentrated under reduced pressure. The crude product was purified by column chromatography (1:20 EtOAc:*n*-hexane) to yield the product as a pale yellow oil (170 mg, 35%). $[\alpha]_D^{22}$ = -129 (*c* 0.5, diethyl ether); **^{1}H NMR** (700 MHz; $CDCl_3$) δ 7.29 – 7.25 (m, 2H, 3-H), 7.19 – 7.16 (m, 3H, 2-H and 4-H), 6.55 (d, *J* = 5.8 Hz, 1H, 1'-H), 3.52 (m, 1H, C<u>H</u>H-OMe), 3.37 (m, 2H, CH<u>H</u>-OMe and 2"-H), 3.36 (s, 3H, OMe), 3.32 – 3.27 (m, 1H, 5"-<u>H</u>H), 2.87 (dd, *J* = 13.4, 6.3 Hz, 1H, 3'-<u>H</u>H), 2.72 (m, 1H, 5"-H<u>H</u>), 2.64 (m, 1H, 2'-H), 2.55 (dd, *J* = 13.4, 8.4 Hz, 1H, 3'H<u>H</u>), 1.97 – 1.83 (m, 3H, 3"-<u>H</u>H and 4"), 1.77 (m, 1H, 3"-H<u>H</u>), 1.02 (d, *J* = 6.8 Hz, 3H, Me); **^{13}C NMR** (176 MHz; $CDCl_3$) δ 143.0, 140.6, 129.5, 128.3, 126.0, 74.8, 63.5, 59.4, 50.4, 41.9, 38.9, 26.7, 22.2, 18.5; ***m/z*** [ESI+] 261 ($[M+H]^+$, 100%), 302 ($[M+CH_3CN+H]^+$, 12%); ***m/z*** [HRMS ESI+] found $[M+H]^+$ 261.1960. $C_{16}H_{25}N_2O$ requires 261.1967.

1.3 Synthesis of racemic 2-methyl-3-phenylpropan-1-amine

2-Methyl-3-phenylpropan-1-amine 23[8]

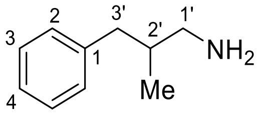

A solution of hexamethyldisilazane (2.3 mL, 11 mmol) in anhydrous THF (10 mL) under argon was cooled to -78 °C and *n*-BuLi (2.5 M in hexanes, 4.0 mL, 10 mmol) was added dropwise. The solution was stirred at 0 °C for 10 mins. In a separate flask, a solution of propionitrile (3.6 mL, 50 mmol) in anhydrous THF (40 mL) under argon was cooled to -78 °C. To this solution, the prepared LiHMDS was added and the reaction stirred for 1 h at -78 °C, then benzyl bromide (1.6 mL, 10 mmol) was added dropwise. The reaction was stirred for 0.5 h at -78 °C, warmed to rt then stirred for 2.5 h. The reaction was concentrated under reduced pressure, diluted with water (100 mL) and extracted with EtOAc (3 × 100 mL). The combined organic layers were dried with $MgSO_4$, filtered, and concentrated under reduced pressure to yield crude 2-methyl-3-phenylpropanenitrile.

A solution of lithium aluminium hydride (2.0 M in THF, 15 mL, 30 mmol) in anhydrous THF (40 mL) under argon was cooled to 0 °C. Crude 2-methyl-3-phenylpropanenitrile in anhydrous THF (10 mL) under argon was added dropwise and the reaction heated to 70 °C for 5 h. The reaction was cooled to 0 °C then 0.74 mL water and 1.0 mL 2 M NaOH was added dropwise and stirred for 1 h at rt. The reaction was dried with Na_2SO_4 and filtered, washing with EtOAc. The resulting filtrate was concentrated under reduced pressure to yield the product as a pale yellow oil (1.1 g). This oil contained

impurities, therefore an internal standard was used to calculate the purity of the compound by NMR (73% by mass) and allow it to be used as a calibration standard for HPLC. **^{1}H NMR** (600 MHz; $CDCl_3$) δ 7.30 – 7.25 (m, 2H, 3-H), 7.25 – 7.13 (m, 3H, 2-H and 4-H), 2.75 – 2.62 (m, 2H, 1'-HH and 3'-HH), 2.52 (dd, J = 12.6, 6.9 Hz, 1H, 1'-HH), 2.38 (dd, J = 13.4, 8.2 Hz, 1H, 3'-HH), 1.83 – 1.72 (m, 1H, 2'-H), 1.40 – 1.27 (m, 2H, NH_2), 0.89 (d, J = 6.6 Hz, 3H, Me); **^{13}C NMR** (151 MHz; $CDCl_3$) δ 141.1, 129.3, 128.4, 125.9, 48.2, 41.1, 38.7, 17.5; ***m/z*** [ESI+] 150 ($[M+H]^+$, 100%), 337 ($[2M+K]^+$, 78%). All spectral data corresponds to those given in the literature.[8]

1.4 Synthesis of PEGs with pendant aldehydes

PEG-8000 supported aldehyde 12

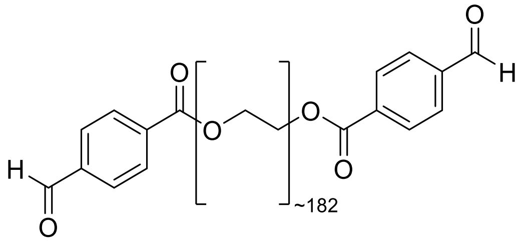

To a solution of PEG-8000 (1.0 g, 0.13 mmol) in dichloromethane (5 mL) under argon was added 4-formylbenzoic acid (75 mg, 0.50 mmol), DCC (52 mg, 0.25 mmol) and DMAP (3 mg, 0.003 mmol) and the solution stirred overnight at room temperature. Diethyl ether (40 mL) was added to precipitate the polymer and the solution cooled to 0 °C. The resulting crystals were filtered, washing with diethyl ether, and dried under reduced pressure to yield the crude product as a white solid (0.92 g, 92%). **Mp** 44 – 48 °C (diethyl ether); **IR** (film) ν_{max}/cm^{-1}: 2883, 2740, 1724, 1095 (C-O); **^{1}H NMR** (700 MHz; $CDCl_3$) δ 10.12 (s, 1H, CHO), 10.08 (s, 1H, CHO), 8.21 (dd, J = 8.2, 1.8 Hz, 4H, Ar-H), 7.95 (dd, J = 8.2, 1.8 Hz, 4H, Ar-H), 4.51 (m, 4H, $PEGOCH_2CH_2OCO$), 3.63 (m, PEG).

PEG-2000 supported aldehyde 13

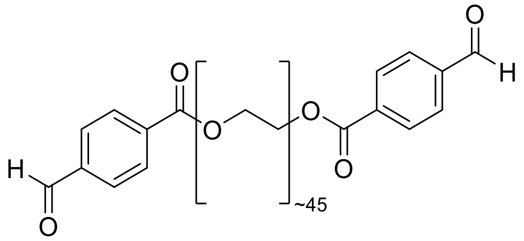

To a solution of PEG-2000 (5.0 g, 2.5 mmol) in dichloromethane (50 mL) under argon was added 4-formylbenzoic acid (1.5 g, 10 mmol, 4 equiv.), DCC (1.0 g, 5.0 mmol, 2 equiv.) and DMAP (60 mg, 0.50 mmol, 0.2 equiv.) and the solution stirred overnight at room temperature. Diethyl ether (200 mL)

was added to precipitate the polymer and the solution cooled to 0 °C. The resulting crystals were filtered, washing with diethyl ether, and the filtrate was concentrated under reduced pressure to yield the crude product as a white solid (4.50 g, 90%). **Mp** 35 – 37 °C (diethyl ether); **IR** (film) ν_{max}/cm^{-1}: 2883, 2741, 1720, 1101; **^{1}H NMR** (600 MHz, $CDCl_3$) δ 10.13 (s, 1H, CHO), 10.08 (s, 1H, CHO), 8.23 (m, 4H, Ar-H), 7.97 (m, 4H, Ar-H), 4.54 – 4.47 (m, 4H, PEGOCH$_2$C$\underline{H_2}$OCO), 3.89 – 3.54 (m, PEG); ***m/z*** [ESI+] 726 [H(OCH_2CH_2)$_{46}$OAr+3H]$^{3+}$, 667 [H(OCH_2CH_2)$_{45}$OH+3H]$^{3+}$, 556 [Ar(OCH_2CH_2)$_{44}$OAr+4H]$^{4+}$.

PEG-1000 supported aldehyde 14

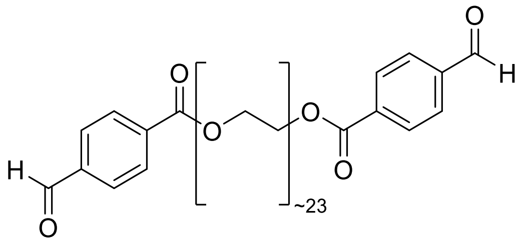

To a solution of PEG-1000 (2.0 g, 2.0 mmol) in dichloromethane (20 mL) under argon was added 4-formylbenzoic acid (1.2 g, 8.0 mmol), DCC (820 mg, 4.0 mmol) and DMAP (48 mg, 0.4 mmol) and the solution stirred overnight at room temperature. Diethyl ether (60 mL) was added to precipitate the polymer and the solution cooled to 0 °C. The resulting crystals were filtered, washing with diethyl ether, and the filtrate was concentrated under reduced pressure to provide a white solid. This was then dissolved in water and filtered. The filtrate was concentrated under reduced pressure to yield the crude product as a sticky off-white solid (1.8 g, 89%). **IR** (film) ν_{max}/cm^{-1}: 2872, 2750, 1720, 1091; **^{1}H NMR** (600 MHz; $CDCl_3$) δ 10.12 (s, 1H, CHO), 10.07 (s, 1H, CHO), 8.22 (dd, *J* = 8.3, 1.9 Hz, 4H, Ar-H), 8.03 – 7.88 (m, 4H, Ar-H), 4.55 – 4.48 (m, 4H, PEGOCH$_2$C$\underline{H_2}$OCO), 3.95 – 3.50 (m, PEG); ***m/z*** [ESI+] 648 [Ar(OCH_2CH_2)$_{23}$OAr+2H]$^{2+}$.

1.5 Control reactions

PEG supported aldehyde control reactions The assay was performed in Eppendorf tubes (500 µL total volume) containing:

Furfurylamine standard: 50 mM furfurylamine in potassium phosphate buffer (100 mM, pH 8.0) in D_2O.

PEG-aldehyde **13** + furfurylamine: 50 mM furfurylamine and 50 mM PEG-2000 supported aldehyde in potassium phosphate buffer (100 mM, pH 8.0) in D_2O shaken at 45 °C and 400 rpm for 24 h.

PEG-aldehyde **13** + isopropylamine: 2.5 M isopropylamine and 50 mM PEG-2000 supported aldehyde in potassium phosphate buffer (100 mM, pH 8.0) in D_2O shaken at 45 °C and 400 rpm for 24 h.

PEG-aldehyde **13**: 50 mM PEG-2000 supported aldehyde in potassium phosphate buffer (100 mM, pH 8.0) in D_2O shaken at 45 °C and 400 rpm for 24 h.

After incubation, the reactions were visualised by NMR, with reference to the solvent peak at 4.79 ppm. There appears to be no reaction between IPA and the PEG-aldehyde, however, there does appear to be some imine formation between the amine product and PEG-aldehyde. As this is a reversible reaction, and the yield increases with the addition of this PEG-aldehyde **13**, it can be assumed that the binding of the hydrazine is favourable enough that the resin is overall beneficial to this reaction.

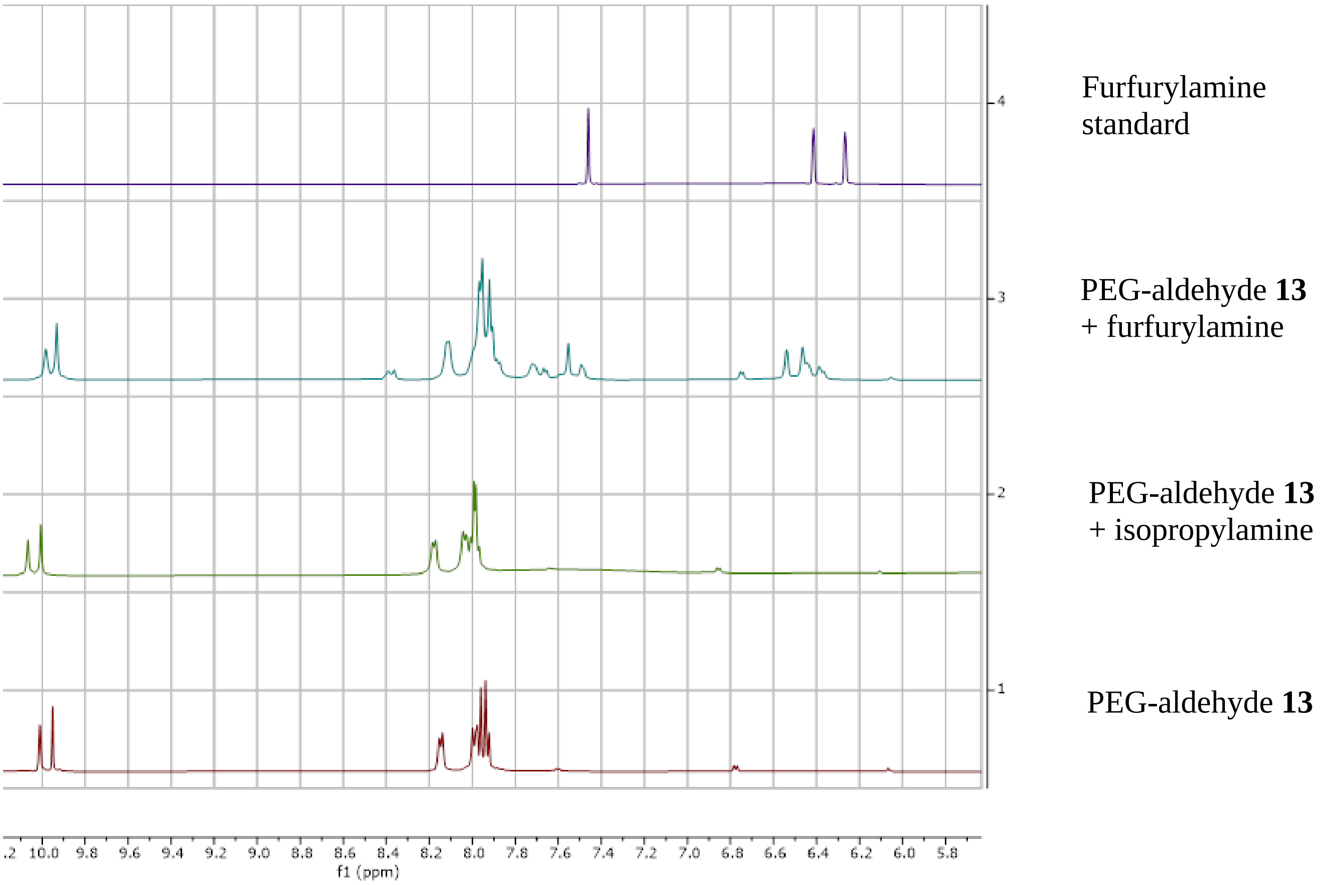

Proportions of hydrazone, aldehyde and amine in a representative reaction

The graph below shows the proportions of hydrazone starting material, aldehyde intermediate and amine product in a representative reaction with hydrazone **4** and in three control reactions: just hydrazone **4**, hydrazone **4** and IPA, hydrazone **4** and PLP. Only the reaction with transaminase gave any amine product. All show small amounts of aldehyde present.

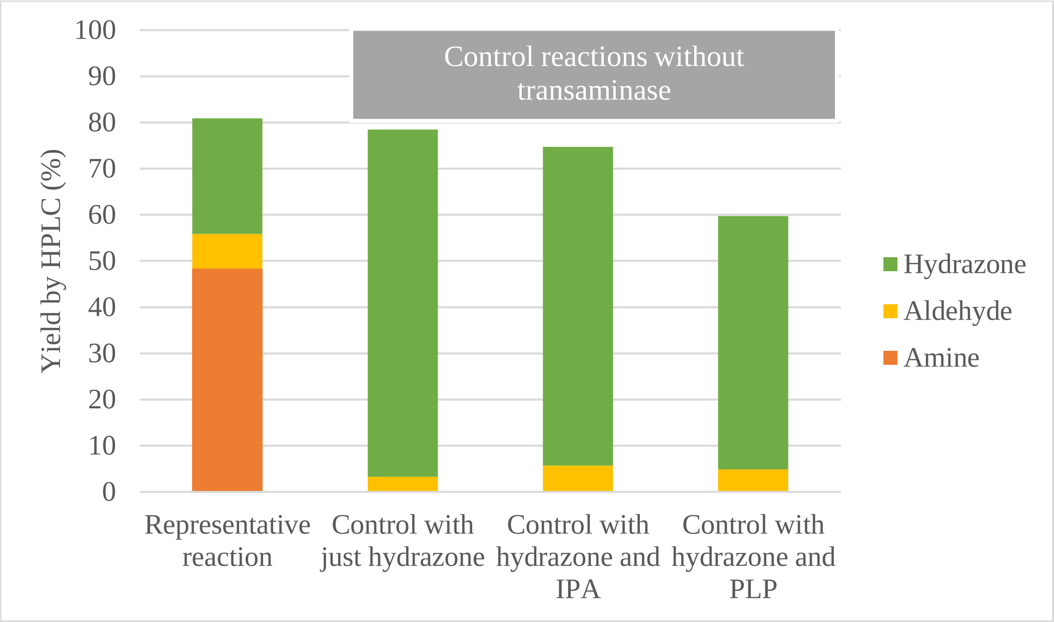

The assay was performed in Eppendorf tubes (500 µL total volume) containing:

Representative reaction: 500 mM IPA, 10 mM PLP, furfuryl hydrazone **4** (10 mM from 100 mM stock in acetonitrile) and crude cell lysate containing the overexpressed TAm (50 µL) in potassium phosphate buffer (100 mM, pH 8.0).

Control with just hydrazone: furfuryl hydrazone **4** (10 mM from 100 mM stock in acetonitrile) in potassium phosphate buffer (100 mM, pH 8.0).

Control with hydrazone and IPA: 500 mM IPA and furfuryl hydrazone **4** (10 mM from 100 mM stock in acetonitrile) in potassium phosphate buffer (100 mM, pH 8.0).

Control with hydrazone and PLP: 10 mM PLP and furfuryl hydrazone **4** (10 mM from 100 mM stock in acetonitrile) in potassium phosphate buffer (100 mM, pH 8.0).

After incubation at 45 °C and 400 rpm for 24 h, the reaction was stopped by the addition of 0.1% TFA in water (100 µL). Denatured protein was removed by centrifugation (4 °C, 12000 rpm, 30 min) and the supernatant diluted with water and analysed by analytical HPLC.

2 Chemical Biology

2.1 General methods

Thermoshakers used were BIOER Mixing Block MB-102 and Stuart Orbital Incubator SI600.

Centrifuges used were Eppendorf Centrifuge 5415R, Eppendorf Centrifuge 5810R and Beckman Coulter Avanti JXN-26.

Autoclaves used were Priorclave TACTROL 2 and Priorclave TACTROL 3. Where sterilisation of waste and media was performed, temperature was held at 121 °C for 30 min.

Sonicators used was Branson Sonifier 150 with microprobe tip at a power of 13 W.

Potassium phosphate buffer (100 mM, pH 8.0): 820 mg K_2HPO_4 and 41 mg KH_2PO_4 made up to 50 mL in distilled water.

Terrific broth (TB) 47.6 g of TB and 4 mL glycerol in 1 L distilled water then autoclaved.

2.2 Protein expression

Overnight cultures (10 mL) of the TAms in *E. coli* from the UCL TAm library were prepared in terrific broth supplemented with kanamycin (50 µg/mL) and incubated overnight at 37 °C. Cells were subcultured using 1% *v/v* inoculum in 2 L shake flasks containing 500 mL of the same supplemented broth at 37 °C and 250 rpm. Transaminases were induced with 1 mM of IPTG when growing in early exponential phase (OD_{600} = 0.6 to 0.8), and the temperature was dropped to 25 °C overnight until harvesting. Cells were harvested by centrifugation and stored at -80 °C. When needed, the cell pellet was resuspended in the appropriate buffer containing 2 mM PLP at a 1:25 volume ratio (1 mL of the resuspension buffer per 25 mL of original cell suspension before harvesting) and sonicated on ice using 10 cycles of 10 s on, 10 s off at 13 W. The sonicated suspension was centrifuged at 12,000 rpm at 4 °C for 45 min to obtain the clarified lysate. The total protein concentration was determined using the Bradford method[9] as 40.0 mg/mL *Cv*-TAm[10], 10.3 mg/mL *Rh*-TAm[11], 12.8 mg/mL *Mv*-TAm[12], 15.1 mg/mL *As*-TAm[13], 23.3 mg/mL *94*-TAm pQR2189[14], 16.9 mg/mL *553*-TAm pQR2191[14], 14.8 mg/mL *3588*-TAm pQR2208[14].

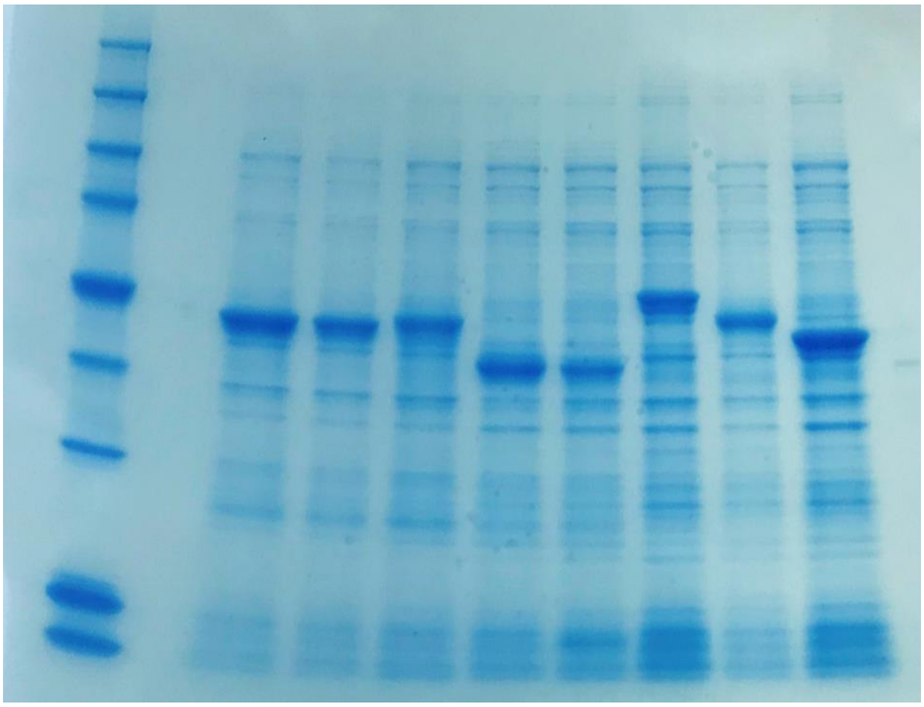

Figure 1: SDS-page gel of *Chromobacterium violaceum* (*Cv*-TAm)[10], *Rhodobacter sphaeroides* (*Rh*-TAm)[11], *Mycobacterium vanbaalenii* (*Mv*-TAm)[12], *Arthrobacter sp.* variant ArRMut11 (*As*-TAm)[13] and three metagenomic enzymes from a domestic drain[14] 94-TAm pQR2189, 553-TAm pQR2191 and 3588-TAm pQR2208.

2.3 Assays

Colorimetric screening. The assay was performed in a 96 well-plate with a total volume of 200 µL containing 2-(4-nitrophenyl)ethan-1-amine hydrochloride (25 mM), amine acceptor (10 mM from 200 mM stock in DMSO), PLP (0.2 mM) and potassium phosphate buffer (100 mM, pH 8.0). The reaction was started by the addition of TAm clarified lysate (10 µL) containing the overexpressed TAm and the reaction was incubated at 30 °C and 500 rpm for 24 h. A positive control was performed with benzaldehyde as the amine acceptor. A negative control was performed without enzyme. An orange/red colouration indicated that the TAms were active towards the selected amine acceptors.

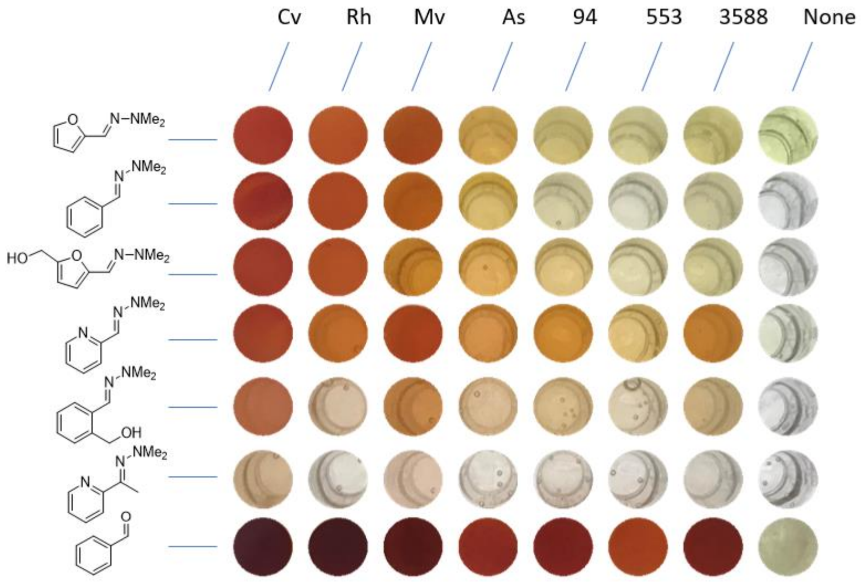

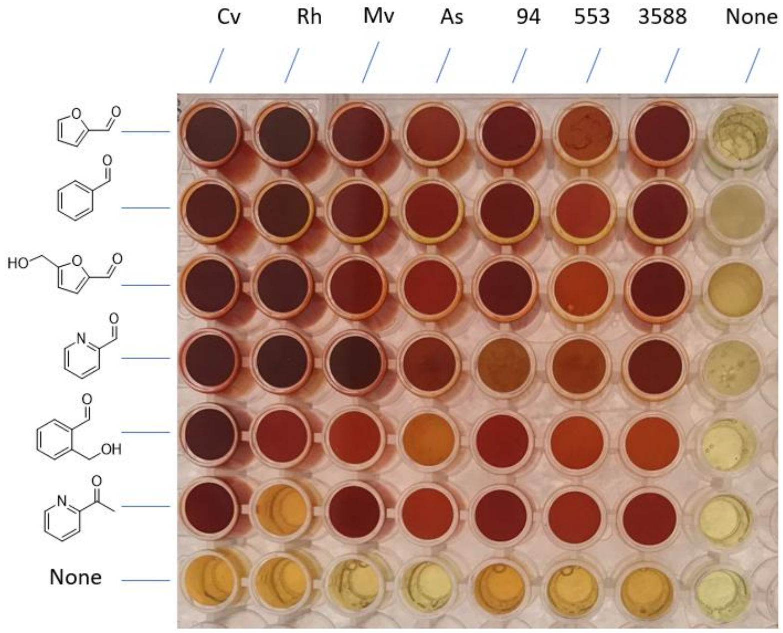

Isopropylamine (IPA) assay. The assay was performed in Eppendorf tubes (500 µL total volume) containing IPA at 500 mM, PLP at the appropriate concentration, PEG-supported aldehyde at the appropriate concentration, potassium phosphate buffer (100 mM, pH 8.0), hydrazone (10 mM from 100 mM stock in acetonitrile) and crude cell lysate (50 µL) containing the overexpressed TAm. After incubation at 45 °C and 400 rpm for 24 h, the reaction was stopped by the addition of 0.1% TFA in water (100 µL). Denatured protein was removed by centrifugation (4 °C, 12000 rpm, 30 min) and the supernatant diluted with water and analysed by analytical HPLC.

Negative controls (no enzyme) were carried out with hydrazone in buffer; hydrazone and IPA in buffer; hydrazone and PLP in buffer; hydrazone, IPA and PLP in buffer. In all reactions there was no amine product detected.

When measuring the enantiomeric excess of the amine product, an acetylation reaction was performed. To 100 µL of the supernatant, pyridine (4.5 µL, 55 equiv.) and acetic anhydride (5.2 µL, 55 equiv.) were added, and the reaction was shaken at 20 °C for 2 h. The reaction was diluted with dichloromethane (100 µL) and extracted with water (2 × 100 µL). The organic layer was concentrated then dissolved in the HPLC mobile phase and analysed by analytical HPLC.

The total protein concentration of the *Cv*-TAm[10] lysate was measured by the Bradford method[9] and found to be 40.0 mg/mL. Analysis of the gel using ImageJ showed that the proportion of *Cv*-TAm was 31% (12.4 mg/mL).

Preparative scale reactions. The reactions were performed in falcon tubes (25-30 mL total volume) containing IPA at 500 mM, PLP at 10 mM, PEG-2000 supported aldehyde at 10 mM, potassium phosphate buffer (100 mM, pH 8.0), hydrazone (10 mM from 100 mM stock in acetonitrile) and crude cell lysate (2.5-3.0 mL) containing the overexpressed TAm. After incubation at 45 °C and 400 rpm for 24 h, a 500 µL aliquot was removed and the reaction was stopped by the addition of 0.1% TFA in water (100 µL) to this aliquot. Denatured protein was removed by centrifugation (4 °C, 12000 rpm, 30 min) and the supernatant diluted with water and analysed by analytical HPLC. Yields of amines **18**, **19** and **23** were determined by analytical HPLC and found to be 38%, 59% and 64%, respectively.

The remaining sample was quenched with 20 mL methanol, acidified with 2 M HCl and extracted with ethyl acetate (3 × 50 mL). The aqueous layer was then basified with 2 M NaOH and extracted with ethyl acetate (3 × 50 mL). The combined organic layers were dried with $MgSO_4$, filtered, and concentrated under reduced pressure. The amine was then further purified by preparative HPLC on a Dionex 580 HPLC system with an Agilent Zorbax 300SB-C18 5 µm 250 x 9.4 mm column.

3-Phenylpropylamine 19[15]

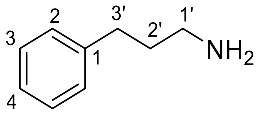

UV detection was measured at 210 nm using a linear gradient 5-50% B over 25 min at 2 mL/min (A = water with 0.1% TFA and B = acetonitrile with 0.1% TFA). Amine **19** eluted with a retention time of 16 mins with a yield of 52%. **^{1}H NMR** (600 MHz, $CDCl_3$) δ 7.31 – 7.07 (m, 5H, Ar-H), 2.90 (m, 2H,

1’-H), 2.65 (t, *J* = 7.1 Hz, 2H, 3’-H), 2.21 (s, 2H, NH_2), 1.96 (m, 2H, 2’-H); **R_f** 0.45 in 20% MeOH, 80% CH_2Cl_2, 0.1% NEt_3. All spectral data corresponds to those given in the literature.[15]

2-Methyl-3-phenylpropan-1-amine 23[8]

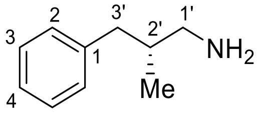

UV detection was measured at 210 nm using a linear gradient 10-16% B over 25 min at 4 mL/min (A = water with 0.1% TFA and B = acetonitrile with 0.1% TFA). Amine **23** eluted with a retention time of 17 mins with a yield of 48%. **^{1}H NMR** (600 MHz; $CDCl_3$) δ 7.30 – 7.12 (m, 5H, Ar-H), 2.77 – 2.68 (m, 2H, 1’-HH and 3’-HH), 2.52 (dd, *J* = 12.4, 7.1 Hz, 1H, 1’-HH), 2.41 (dd, *J* = 13.5, 8.2 Hz, 1H, 3’-HH), 1.94 – 1.86 (m, 1H, 2’-H), 0.92 (d, *J* = 6.7 Hz, 3H, Me); **^{13}C NMR** (151 MHz; $CDCl_3$) δ 138.7, 129.1, 128.8, 126.7, 45.5, 40.6, 33.8, 16.9; **R_f** 0.42 in 20% MeOH, 80% CH_2Cl_2, 0.1% NEt_3. All spectral data corresponds to those given in the literature.[8]

3 Analytical HPLC

Achiral HPLC was performed on an Agilent 1260 Infinity system using an ACE 5-C18-AR column (150 × 4.6 mm).

Chiral HPLC was performed on an Agilent 1260 Infinity system using a Chiralpak AD-H column (250 × 4.6 mm).

Furfurylamine **11**

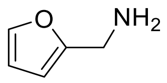

The concentration of furfurylamine was determined with UV detection at 210 nm using a linear gradient 3-30% B over 10 min at 1 mL/min (A = water with 0.1% TFA and B = acetonitrile). The furfurylamine eluted at a retention time of 3.3 min. Results were verified in duplicate or triplicate.

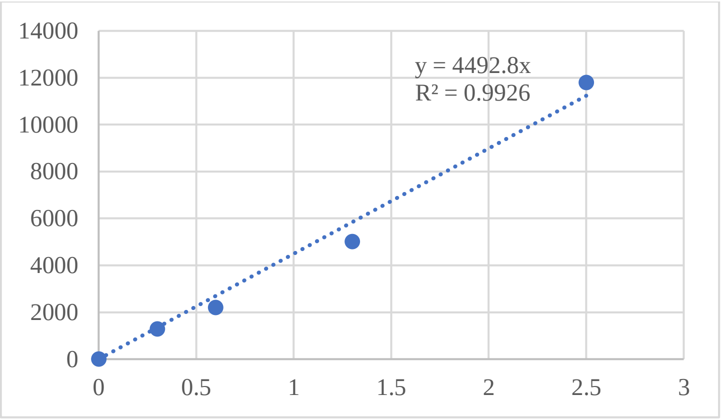

Benzylamine **17**

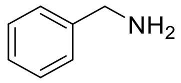

The concentration of benzylamine was determined with UV detection at 210 nm using a linear gradient 5-95% B over 15 min at 1 mL/min (A = water with 0.1% TFA and B = acetonitrile). The benzylamine eluted at a retention time of 5.3 min. Results were verified in duplicate or triplicate.

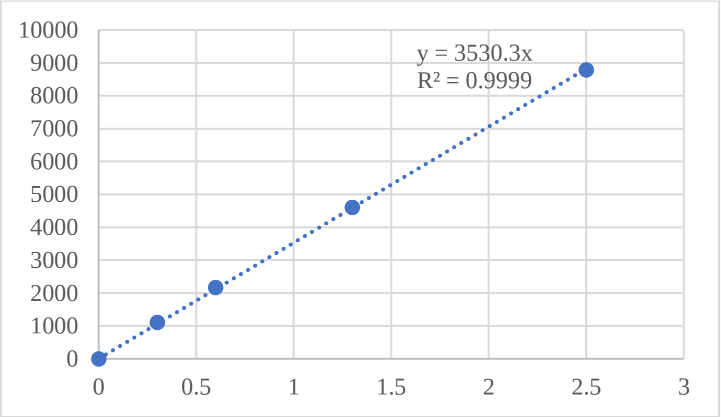

2-Phenylethylamine **18**

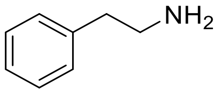

The concentration of 2-phenylethylamine was determined with UV detection at 210 nm using a linear gradient 5-95% B over 15 min at 1 mL/min (A = water with 0.1% TFA and B = acetonitrile). The 2-phenylethylamine eluted at a retention time of 6.4 min. Results were verified in duplicate or triplicate.

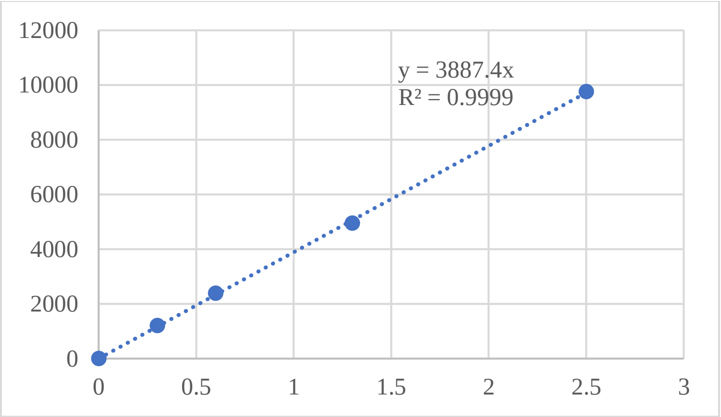

3-Phenylpropylamine **19**

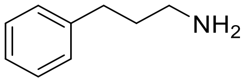

The concentration of 3-phenylpropylamine was determined with UV detection at 210 nm using a linear gradient 5-95% B over 15 min at 1 mL/min (A = water with 0.1% TFA and B = acetonitrile). The 3-phenylpropylamine eluted at a retention time of 7.2 min. Results were verified in duplicate or triplicate.

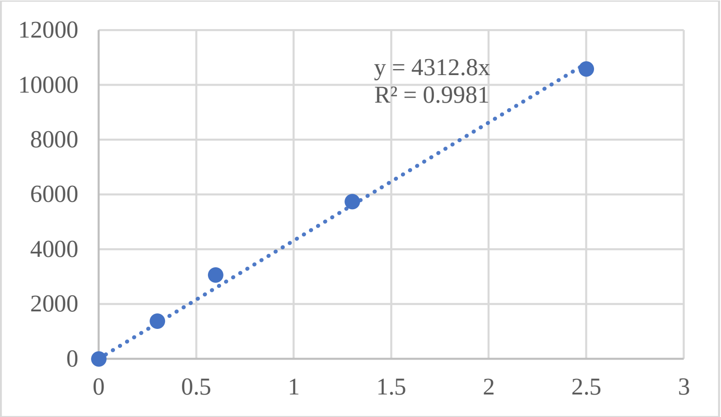

2-Methyl-3-phenyl-propylamine **23**

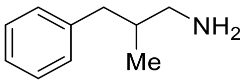

The concentration of 2-methyl-3-phenyl-propylamine was determined with UV detection at 210 nm using a linear gradient 10-20% B over 30 min at 1 mL/min (A = water with 0.1% TFA and B = acetonitrile). The 2-methyl-3-phenyl-propylamine eluted at a retention time of 18.7 min. Results were verified in duplicate or triplicate.

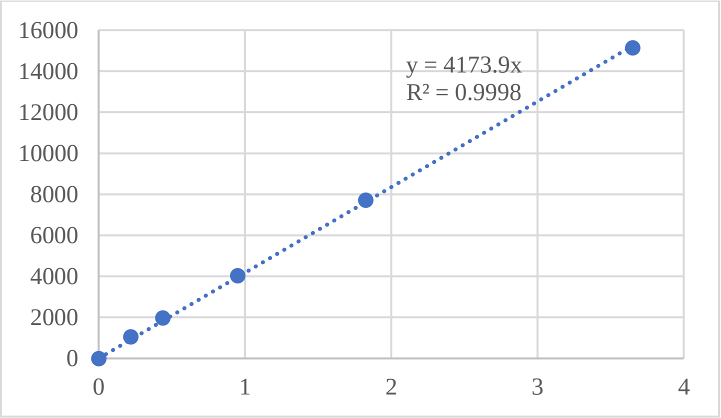

N-(2-Methyl-3-phenylpropyl)acetamide

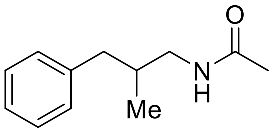

The enantiomeric excess of *N*-(2-methyl-3-phenylpropyl)acetamide was determined with UV detection at 210 nm using an isocratic mobile phase of 5:95 isopropanol:*n*-hexane over 20 min at 1 mL/min. The *N*-(2-methyl-3-phenylpropyl)acetamide eluted at a retention time of 14.9 and 16.0 min. The stereochemistry of the product was assigned according to the literature.[16]

Furfural

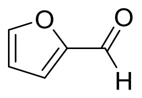

The concentration of furan-2-carbaldehyde was determined with UV detection at 250 nm using an isocratic mobile phase of 3% B over 15 min at 1 mL/min (A = water with 0.1% TFA and B = acetonitrile). The furan-2-carbaldehyde eluted at a retention time of 8.5 min.

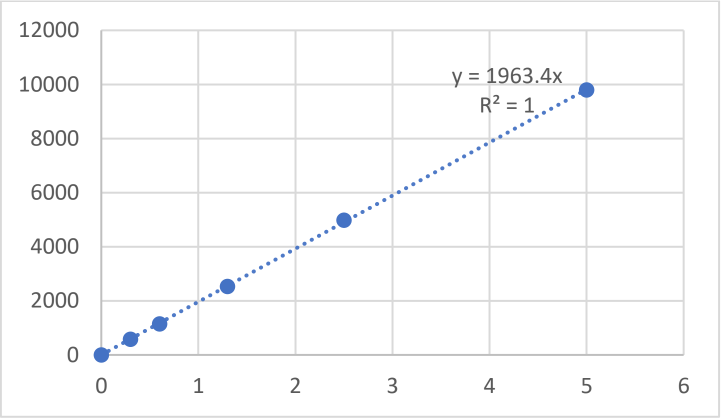

2-(Furan-2-ylmethylene)-1,1-dimethylhydrazine **4**

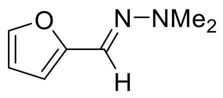

The concentration of 2-(furan-2-ylmethylene)-1,1-dimethylhydrazine was determined with UV detection at 210 nm using a linear gradient of 3-30% B over 15 min at 1 mL/min (A = water with 0.1% TFA and B = acetonitrile). The 2-(furan-2-ylmethylene)-1,1-dimethylhydrazine eluted at a retention time of 9.9 min.

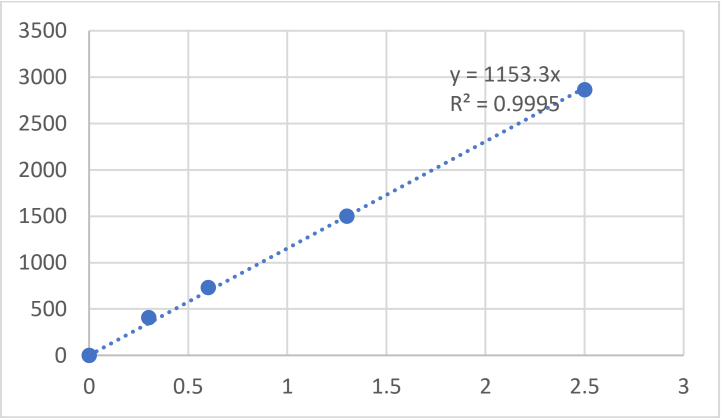

4 NMR spectra

4

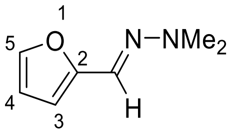

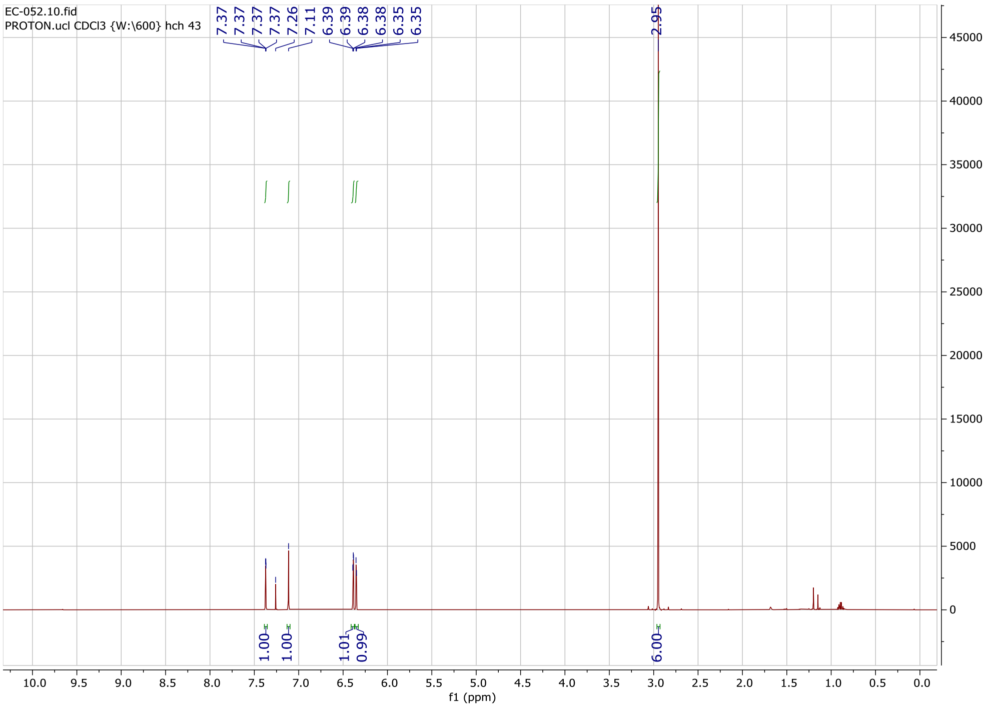

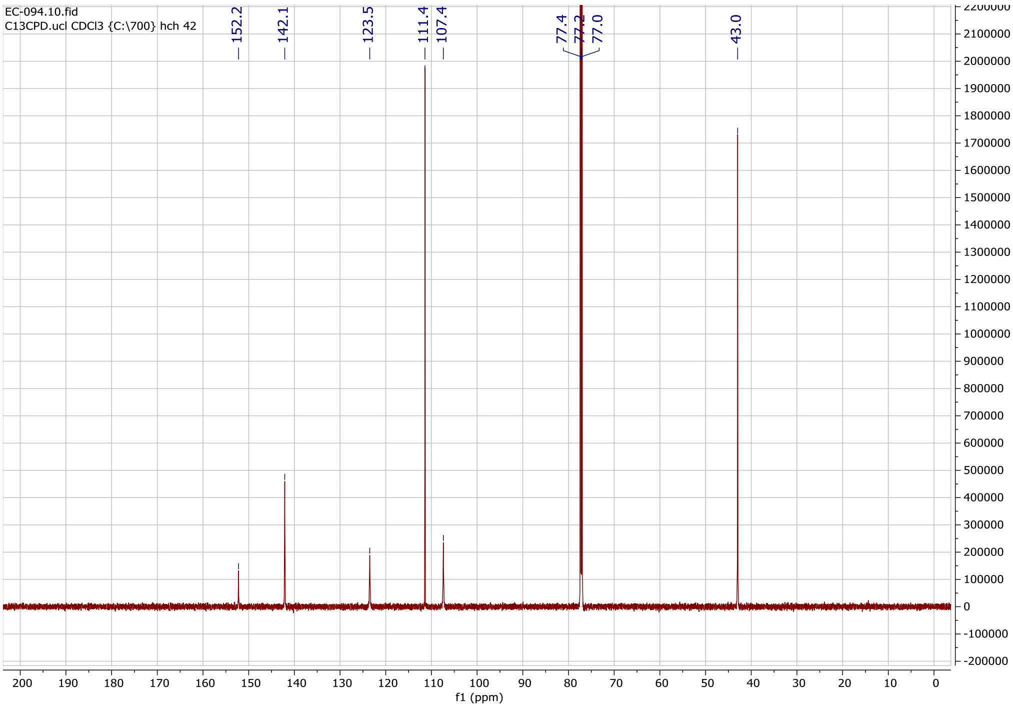

5

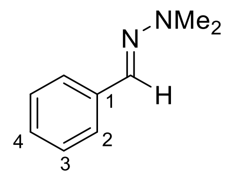
NMe2
N
H
1
2
3
4

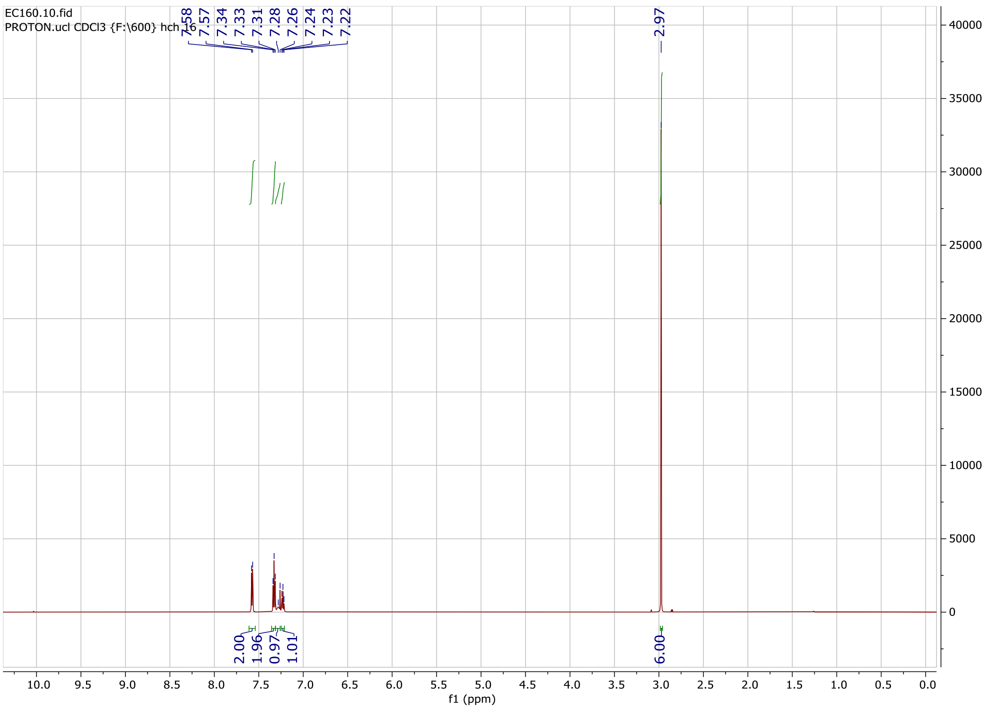
EC160.10.fid
PROTON.ucl CDCl3 {F:\600} hch 16
7.58
7.57
7.34
7.33
7.31
7.28
7.26
7.24
7.23
7.22
2.97
2.00
1.96
0.97
1.01
6.00
f1 (ppm)

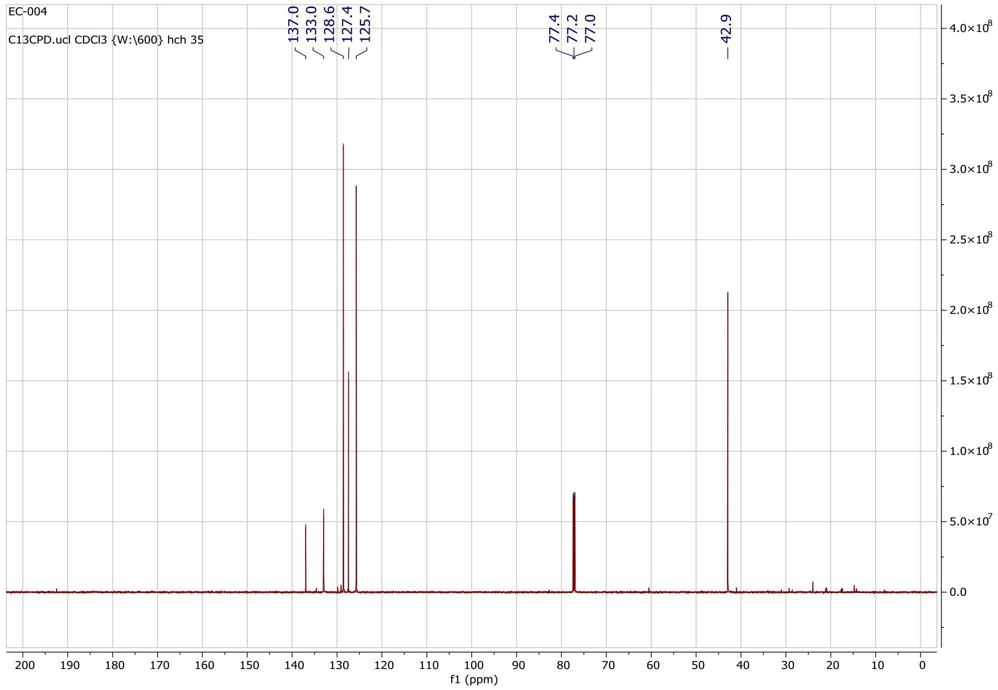
EC-004
C13CPD.ucl CDCl3 {W:\600} hch 35
137.0
133.0
128.6
127.4
125.7
77.4
77.2
77.0
42.9
f1 (ppm)

6

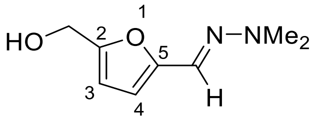
HO
N−NMe2
H
1
2
3
4
5

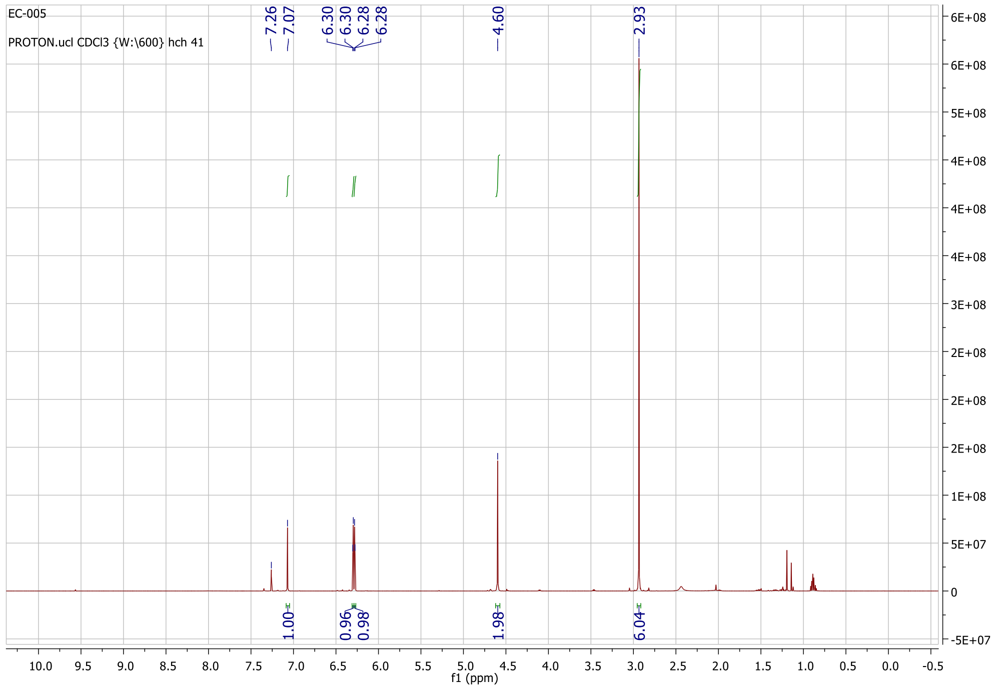
EC-005
PROTON.ucl CDCl3 {W:\600} hch 41
7.26
7.07
6.30
6.30
6.28
6.28
4.60
2.93
1.00
0.96
0.98
1.98
6.04
f1 (ppm)

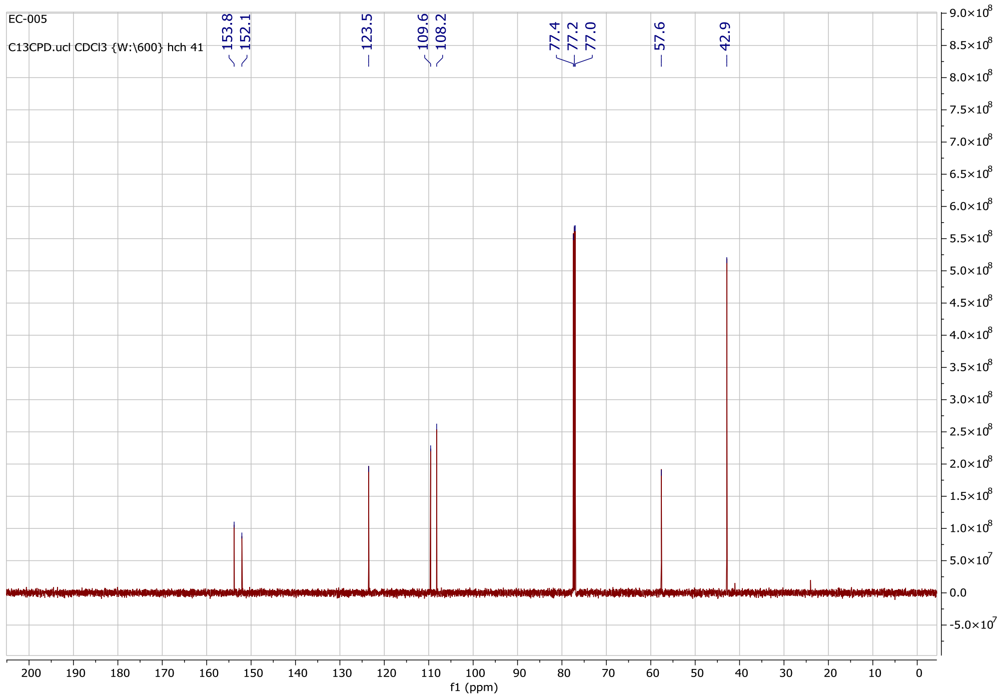
EC-005
C13CPD.ucl CDCl3 {W:\600} hch 41
153.8
152.1
123.5
109.6
108.2
77.4
77.2
77.0
57.6
42.9
f1 (ppm)

7

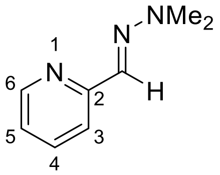
NMe2
N
1
N
6
2
H
5
3
4

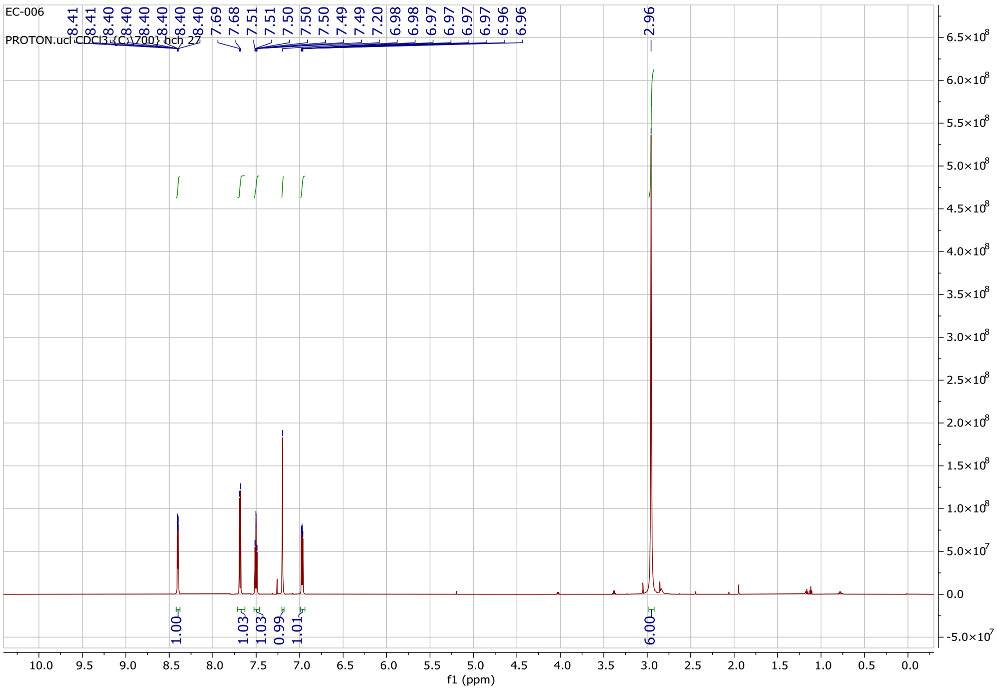
EC-006
PROTON.ucl CDCl3 {C:\700} hch 27
8.41
8.41
8.40
8.40
8.40
8.40
8.40
7.69
7.68
7.51
7.51
7.50
7.50
7.50
7.49
7.49
7.20
6.98
6.98
6.97
6.97
6.97
6.97
6.96
6.96
2.96
1.00
1.03
1.03
0.99
1.01
6.00
f1 (ppm)

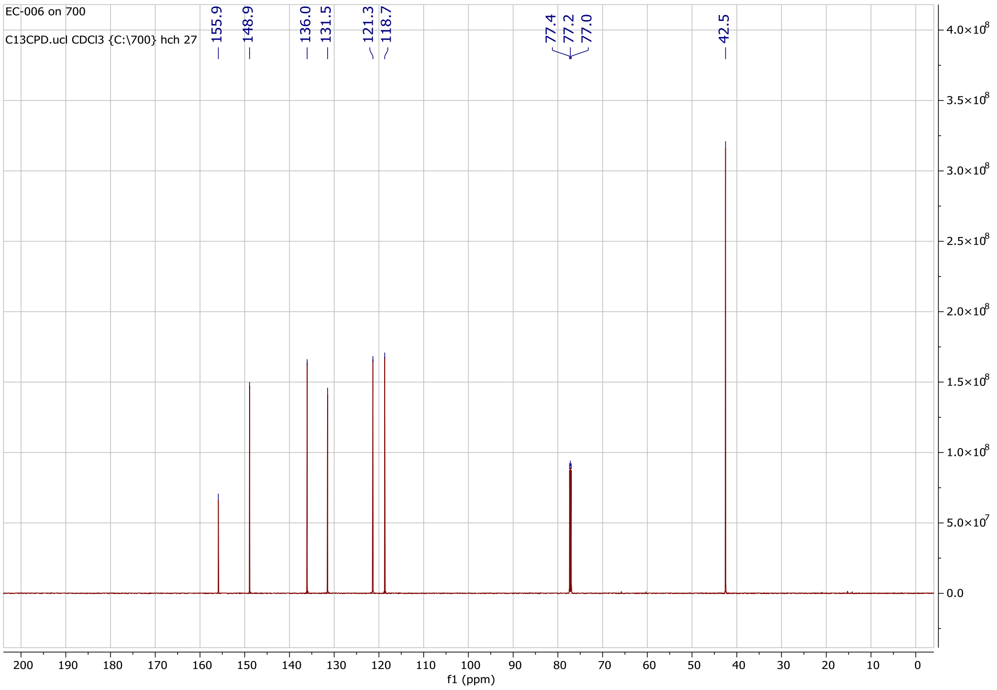
EC-006 on 700
C13CPD.ucl CDCl3 {C:\700} hch 27
155.9
148.9
136.0
131.5
121.3
118.7
77.4
77.2
77.0
42.5
f1 (ppm)

8a

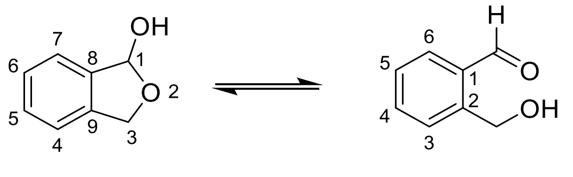

OH
1
2
3
4
5
6
7
8
9
O
H
O
OH
I
II

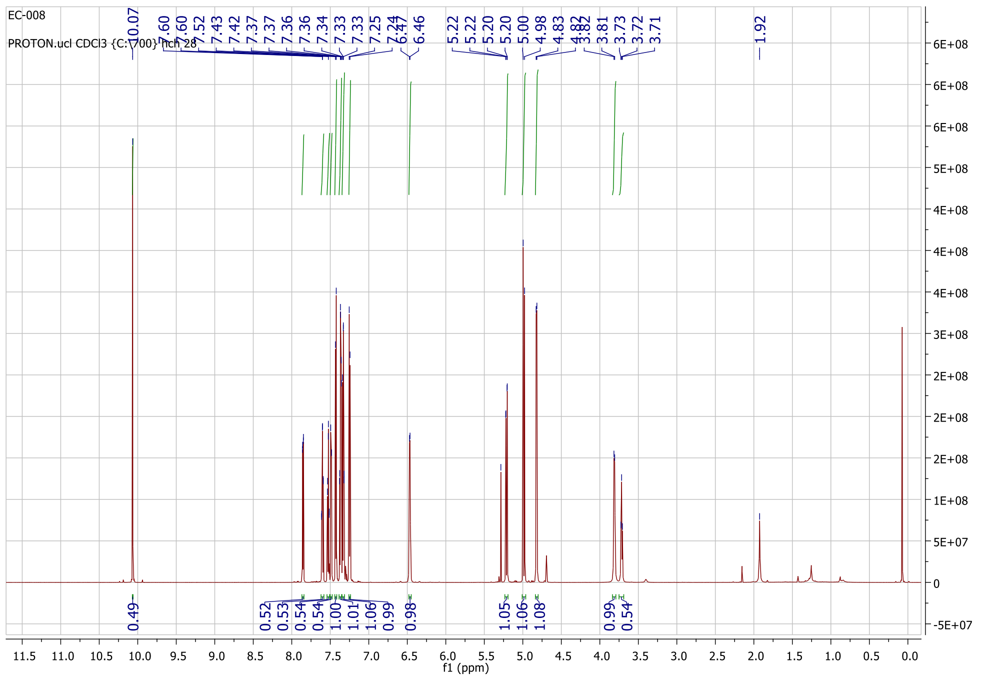

EC-008
PROTON.ucl CDCl3 {C:\700} hch 28
f1 (ppm)

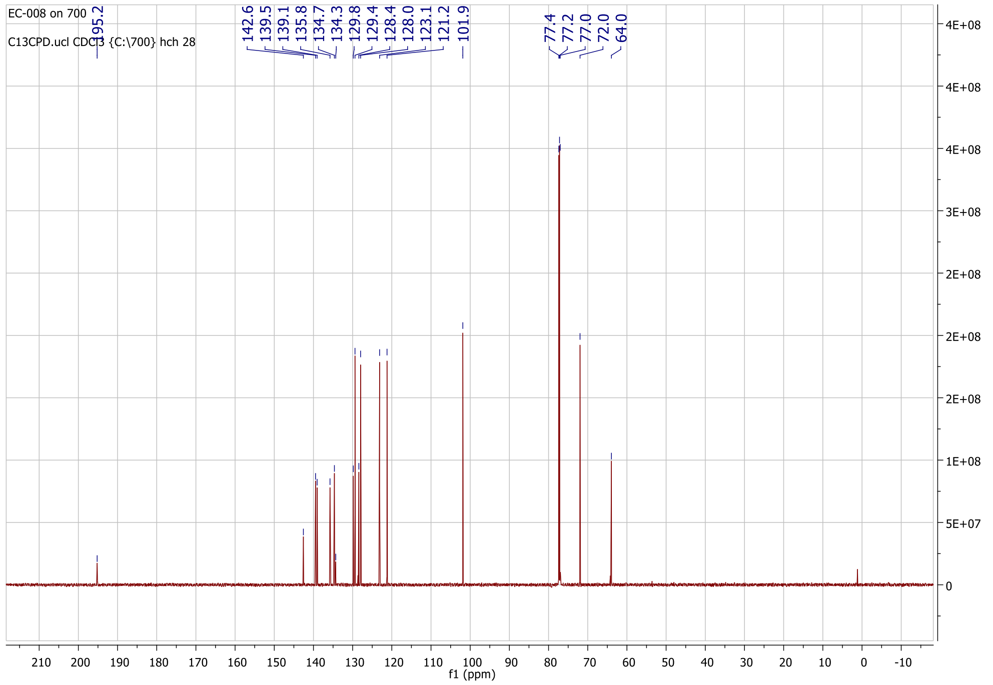

EC-008 on 700
C13CPD.ucl CDCl3 {C:\700} hch 28
f1 (ppm)

8

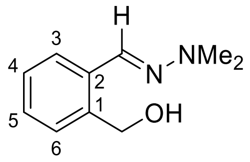
H
NMe2
N
OH
3
4
5
6
2
1

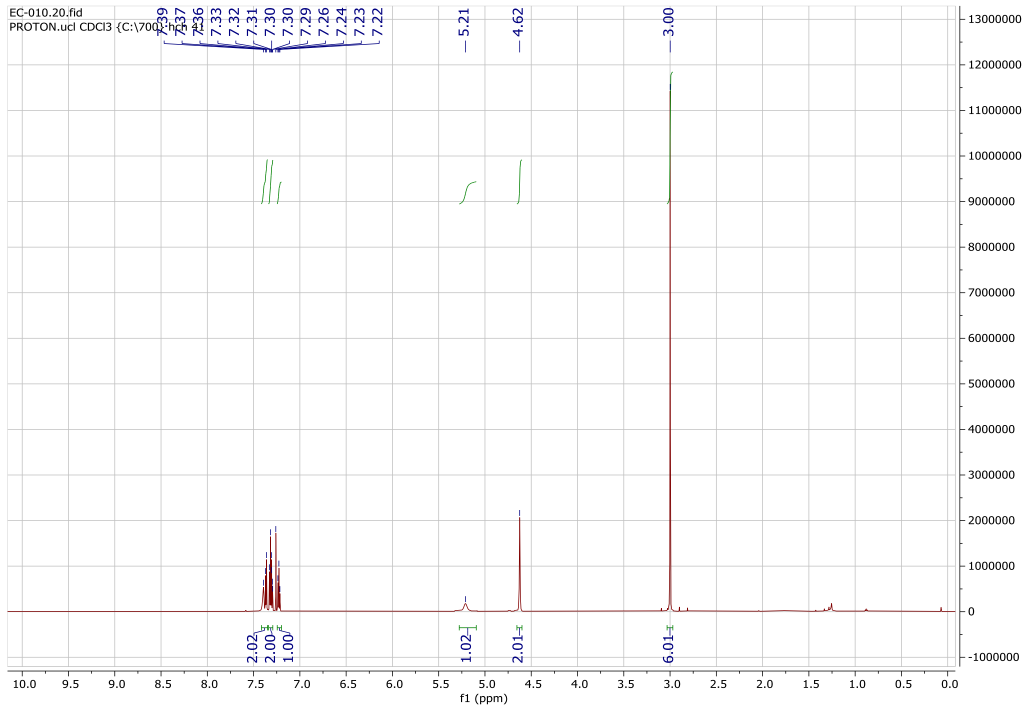
EC-010.20.fid
PROTON.ucl CDCl3 {C:\700} hch 41
7.39
7.37
7.36
7.33
7.32
7.31
7.30
7.30
7.29
7.26
7.24
7.23
7.22
5.21
4.62
3.00
2.02
2.00
1.00
1.02
2.01
6.01
f1 (ppm)

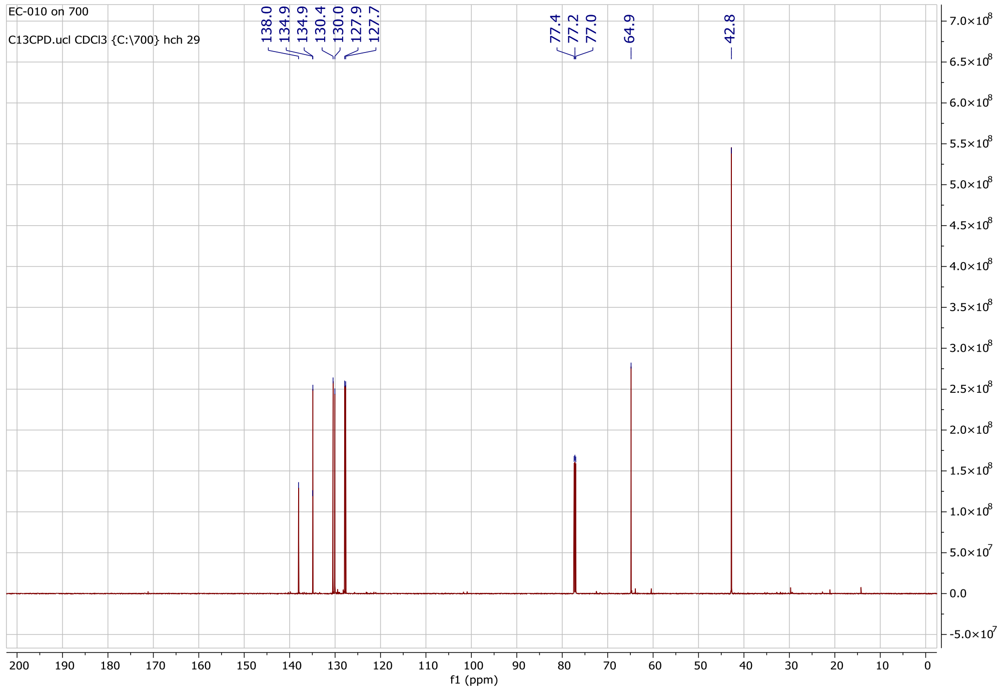
EC-010 on 700
C13CPD.ucl CDCl3 {C:\700} hch 29
138.0
134.9
134.9
130.4
130.0
127.9
127.7
77.4
77.2
77.0
64.9
42.8
f1 (ppm)

9

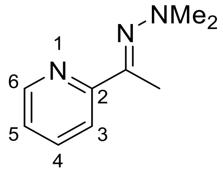
NMe2
N
1
N
6
2
5
3
4

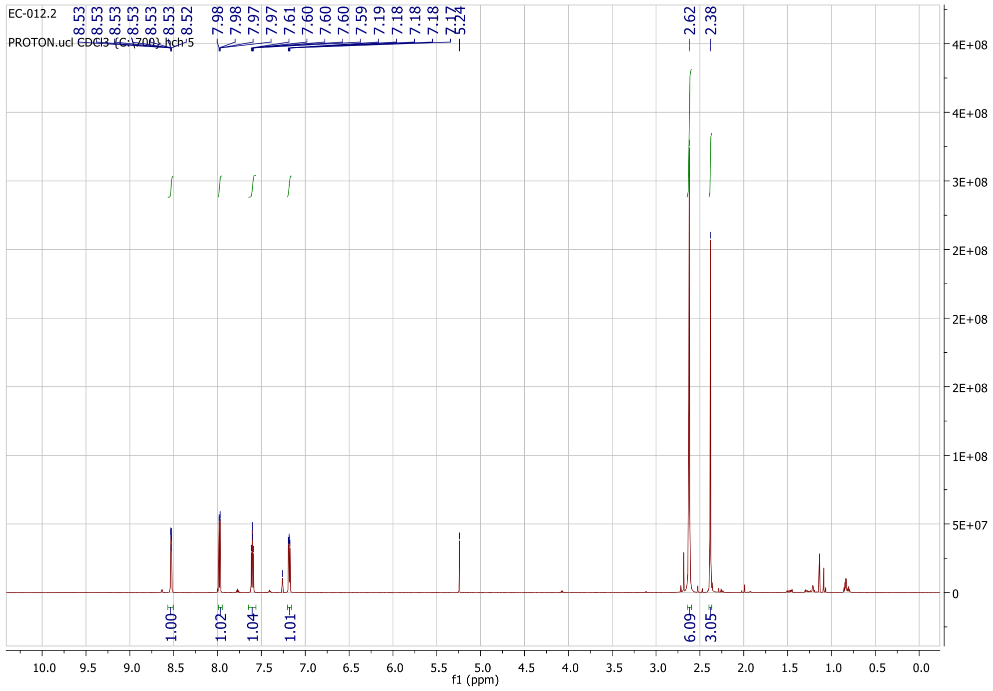
EC-012.2
PROTON.ucl CDCl3 {C:\700} hch 5
8.53
8.53
8.53
8.53
8.53
8.53
8.52
7.98
7.98
7.97
7.97
7.61
7.60
7.60
7.59
7.19
7.18
7.18
7.18
7.17
5.24
2.62
2.38
1.00
1.02
1.04
1.01
6.09
3.05
f1 (ppm)

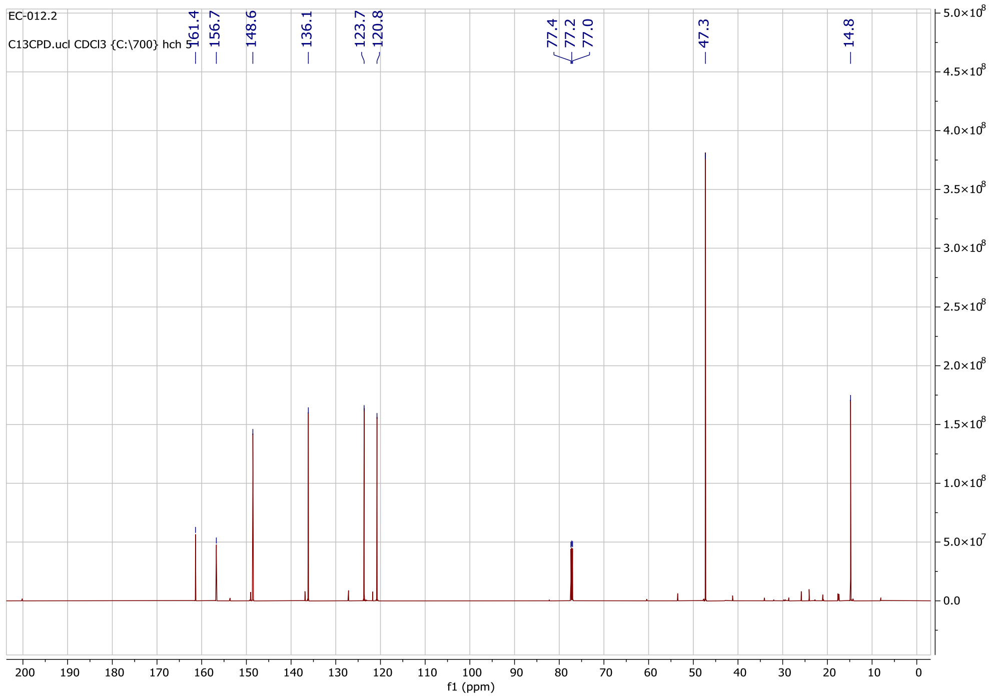
EC-012.2
C13CPD.ucl CDCl3 {C:\700} hch 5
161.4
156.7
148.6
136.1
123.7
120.8
77.4
77.2
77.0
47.3
14.8
f1 (ppm)

15

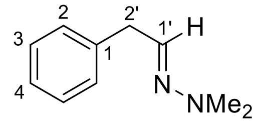
2
2'
1'
H
3
1
N
4
NMe2

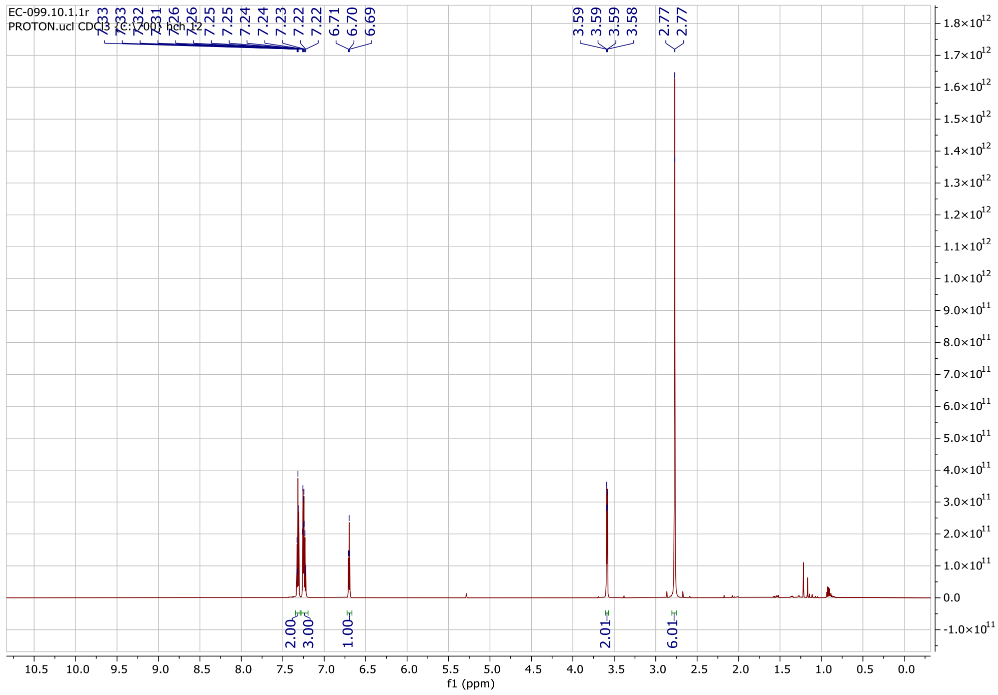
EC-099.10.1.1r
PROTON.ucl CDCl3 {C:\700} hch 12
f1 (ppm)

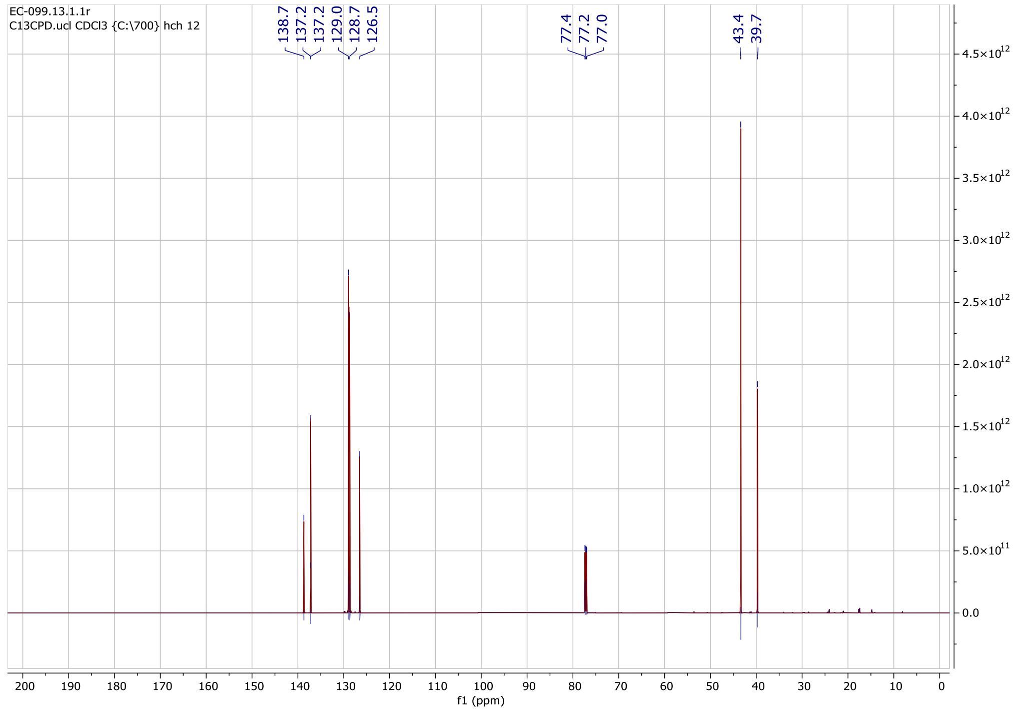
EC-099.13.1.1r
C13CPD.ucl CDCl3 {C:\700} hch 12
138.7
137.2
137.2
129.0
128.7
126.5
77.4
77.2
77.0
43.4
39.7
f1 (ppm)

16

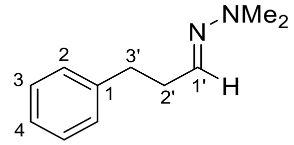
N
NMe2
H
2
3'
3
1
2'
1'
4

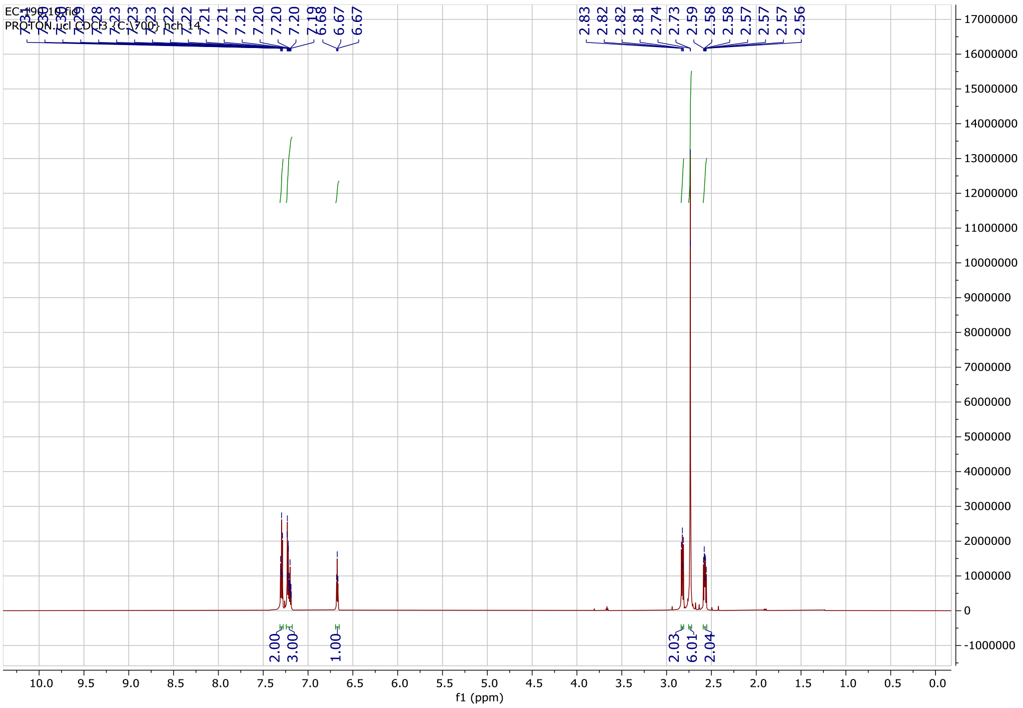
6.68
6.67
6.67
2.83
2.82
2.81
2.74
2.73
2.59
2.58
2.57
2.57
2.56
2.00
3.00
1.00
2.03
6.01
2.04
f1 (ppm)

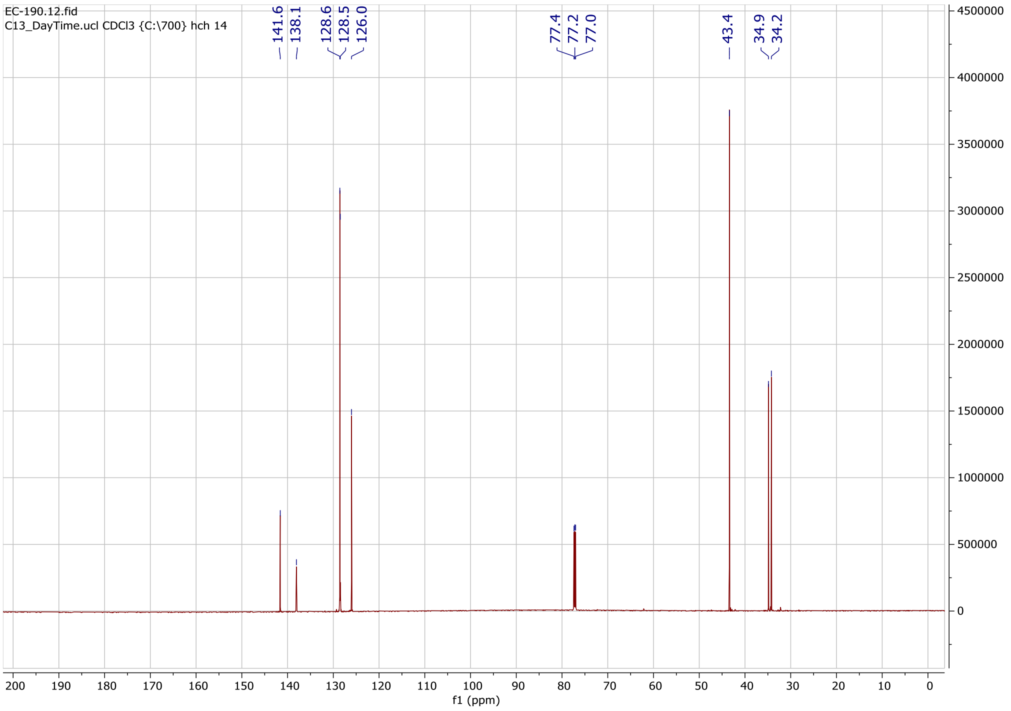
EC-190.12.fid
C13_DayTime.ucl CDCl3 {C:\700} hch 14
141.6
138.1
128.6
128.5
126.0
77.4
77.2
77.0
43.4
34.9
34.2
f1 (ppm)

22

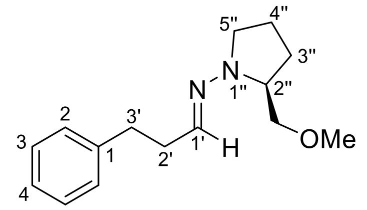

5"
4"
3"
N
N
1"
2"
2
3'
1'
3
1
2'
H
OMe
4

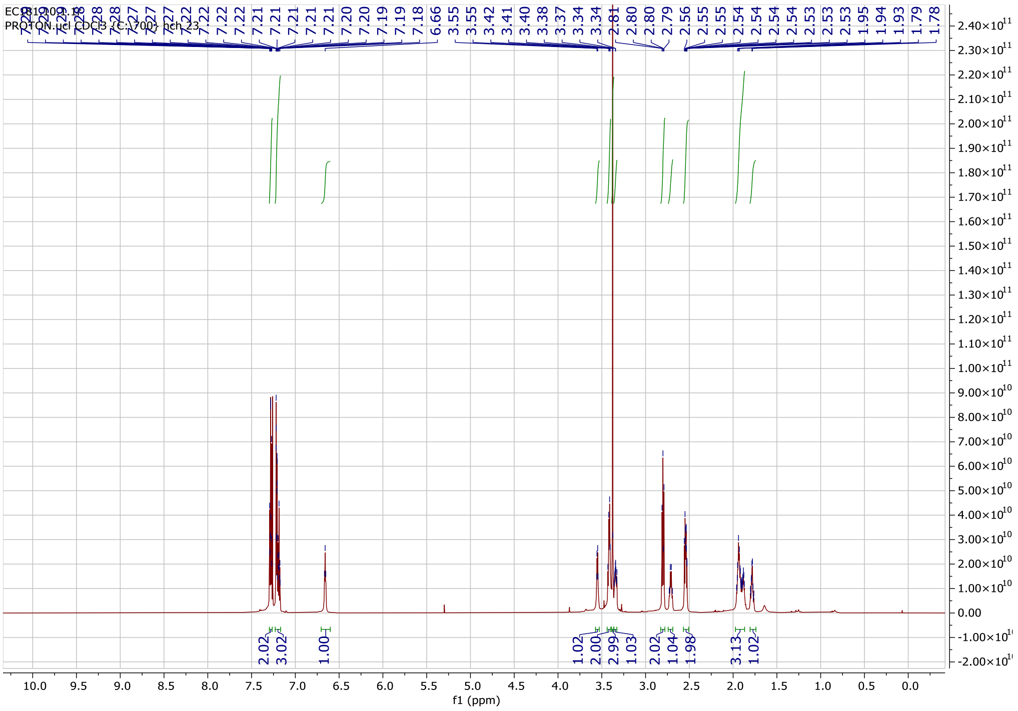

PROTON.ucl CDCl3 {C:\700} hch 23
f1 (ppm)

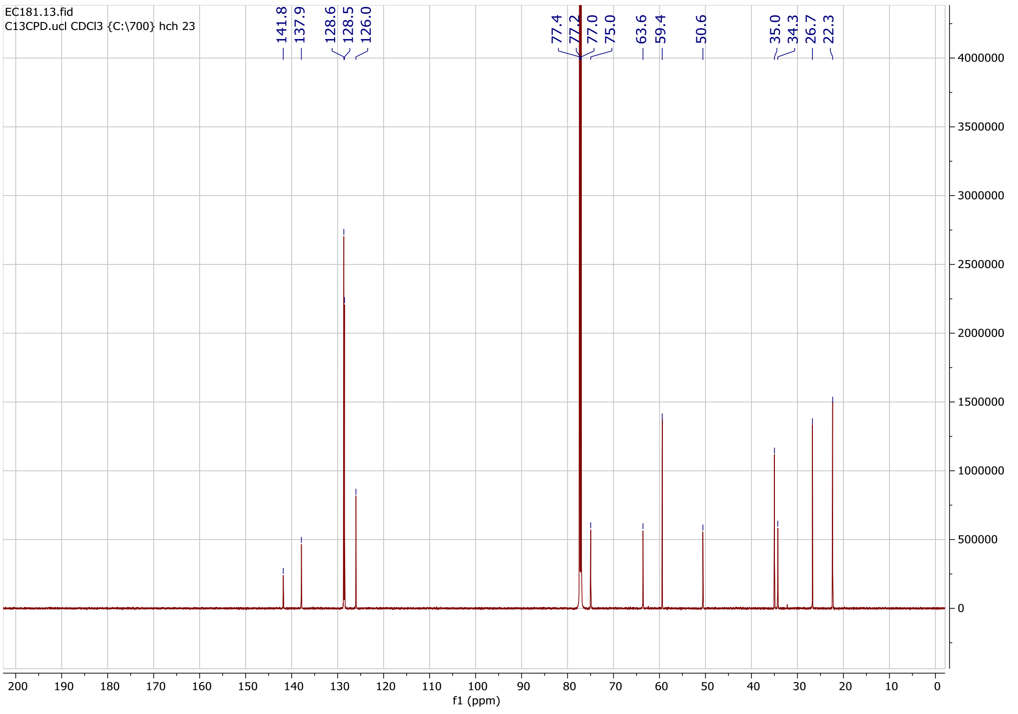

EC181.13.fid
C13CPD.ucl CDCl3 {C:\700} hch 23
141.8
137.9
128.6
128.5
126.0
77.4
77.2
77.0
75.0
63.6
59.4
50.6
35.0
34.3
26.7
22.3
f1 (ppm)

21

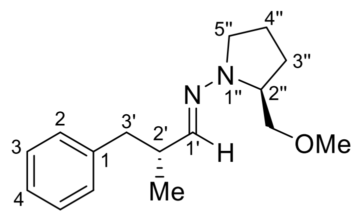
5"
4"
3"
N
N
1"
2"
2
3'
2'
1'
3
1
H
OMe
4
Me

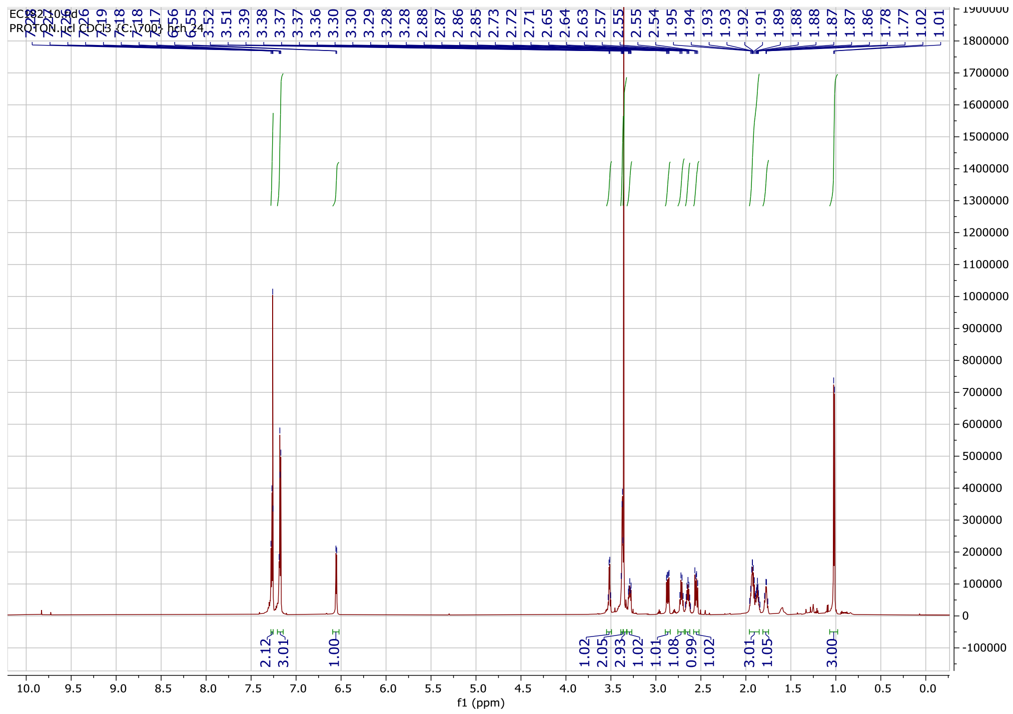
PROTON.ucl CDCl3 {C:\700} hch 24
f1 (ppm)

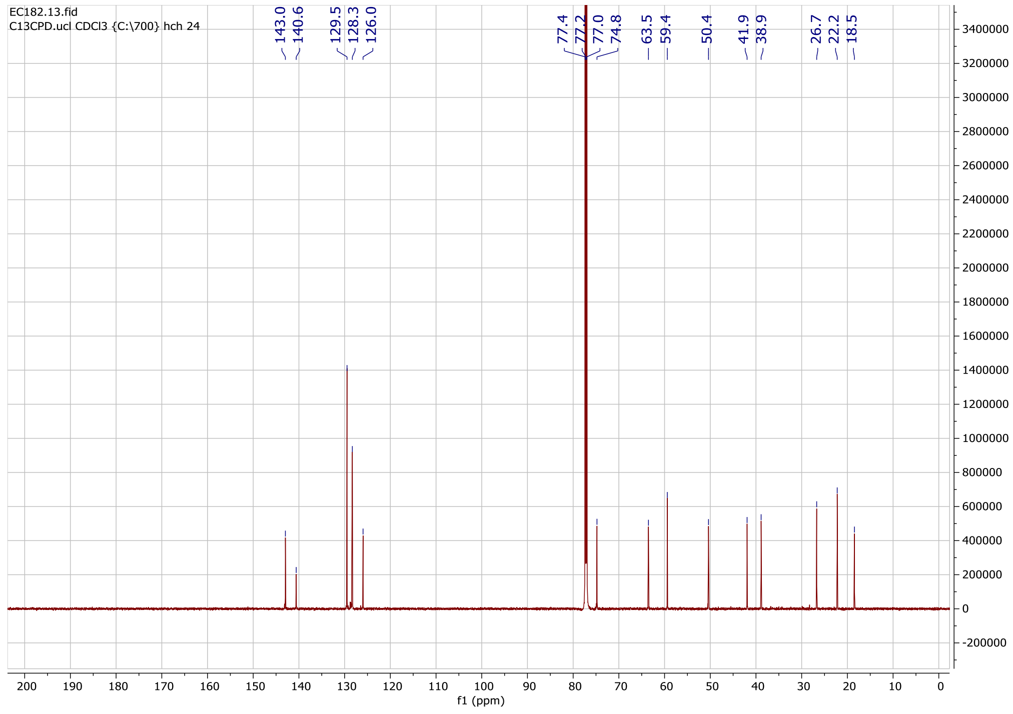
EC182.13.fid
C13CPD.ucl CDCl3 {C:\700} hch 24
143.0
140.6
129.5
128.3
126.0
77.4
77.2
77.0
74.8
63.5
59.4
50.4
41.9
38.9
26.7
22.2
18.5
f1 (ppm)

23

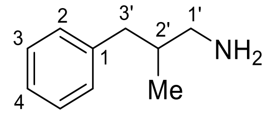
2
3'
1'
3
2'
1
NH2
Me
4

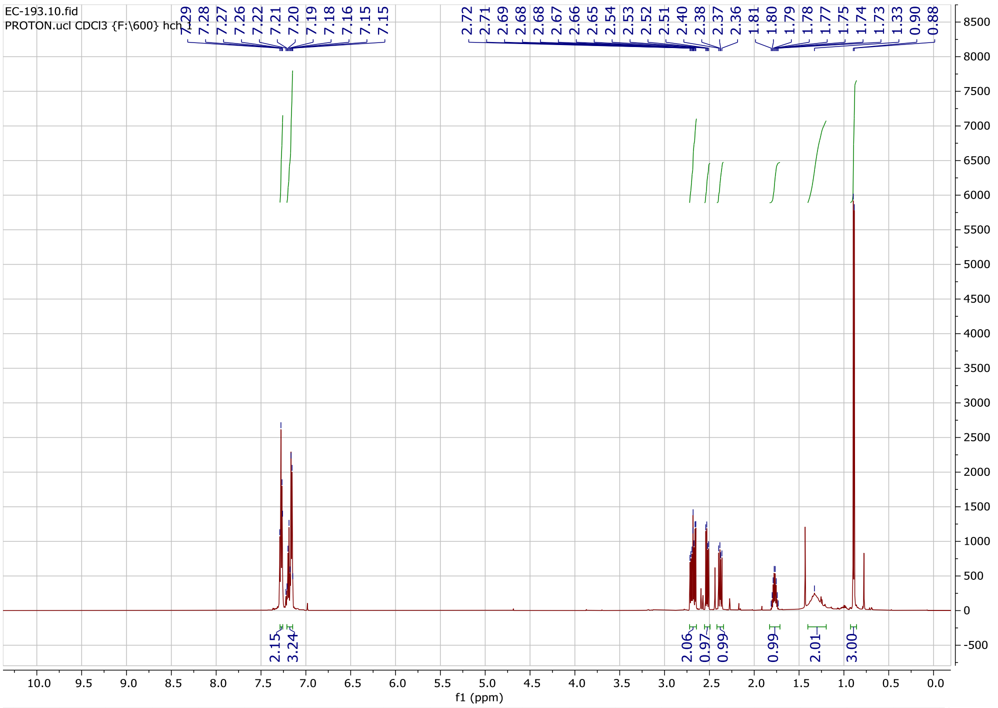
EC-193.10.fid
PROTON.ucl CDCl3 {F:\600} hch 1
7.29 7.28 7.27 7.26 7.22 7.21 7.20 7.19 7.18 7.16 7.15 7.15
2.72 2.71 2.69 2.68 2.68 2.67 2.66 2.65 2.54 2.53 2.52 2.51 2.40 2.38 2.37 2.36
1.81 1.80 1.79 1.78 1.77 1.75 1.74 1.73 1.33 0.90 0.88
2.15 3.24 2.06 0.97 0.99 0.99 2.01 3.00
f1 (ppm)

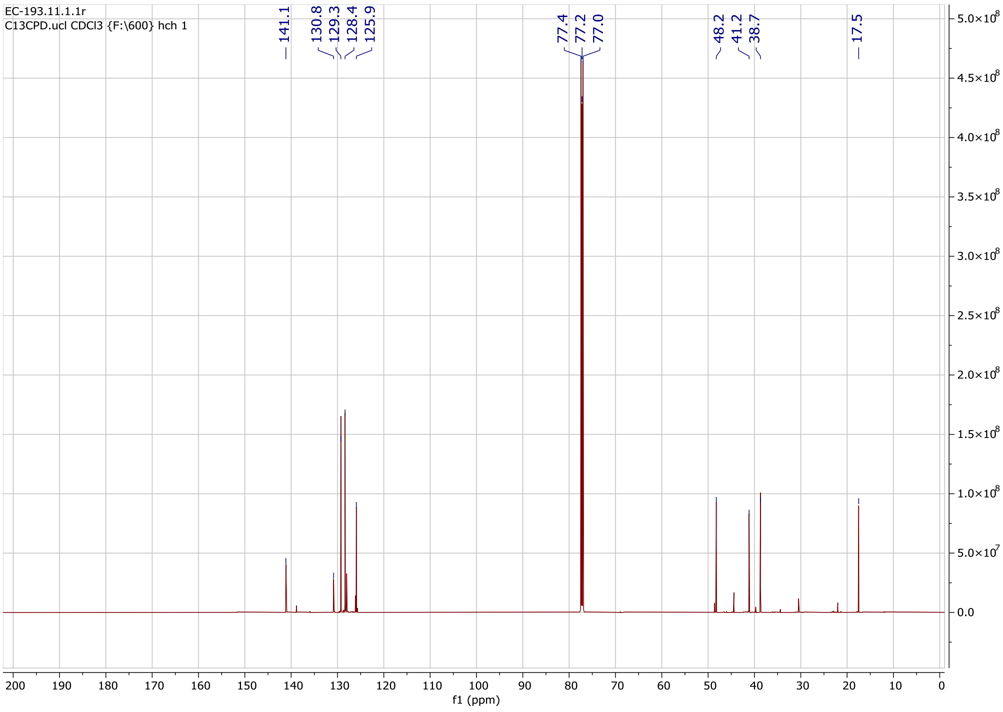
EC-193.11.1.1r
C13CPD.ucl CDCl3 {F:\600} hch 1
141.1
130.8 129.3 128.4 125.9
77.4 77.2 77.0
48.2 41.2 38.7
17.5
f1 (ppm)

12

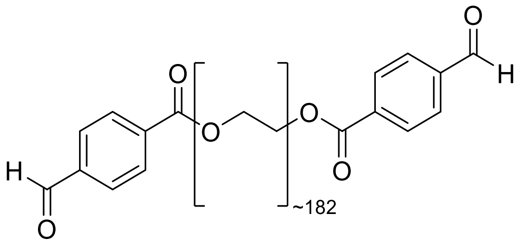

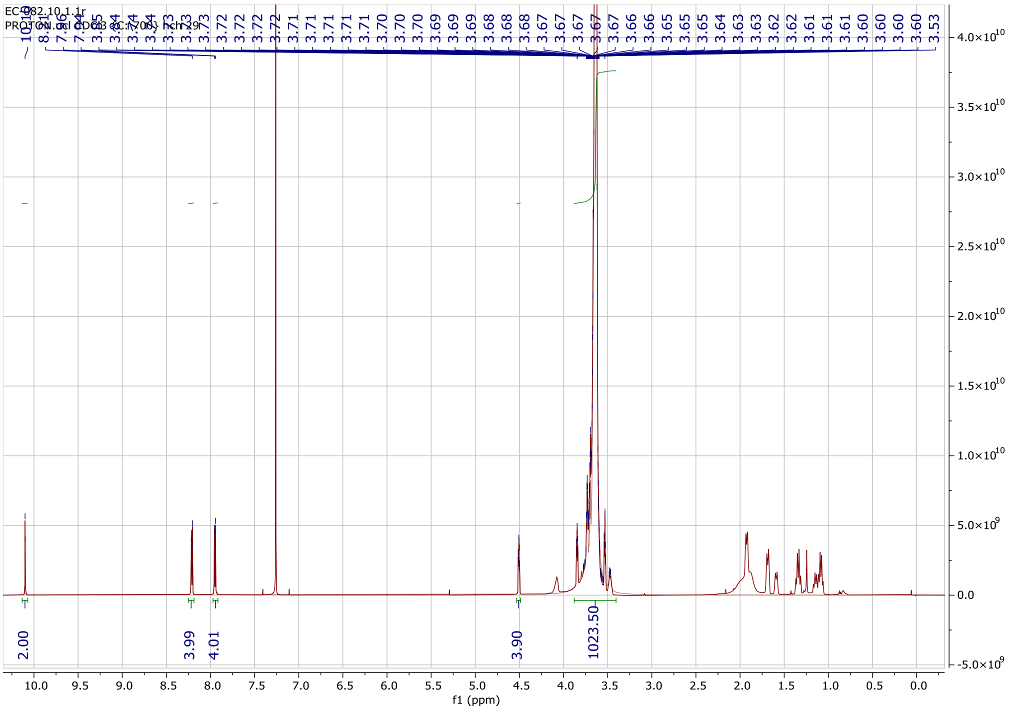

13

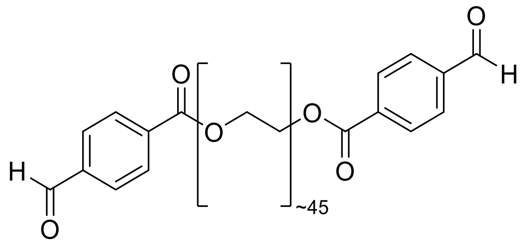

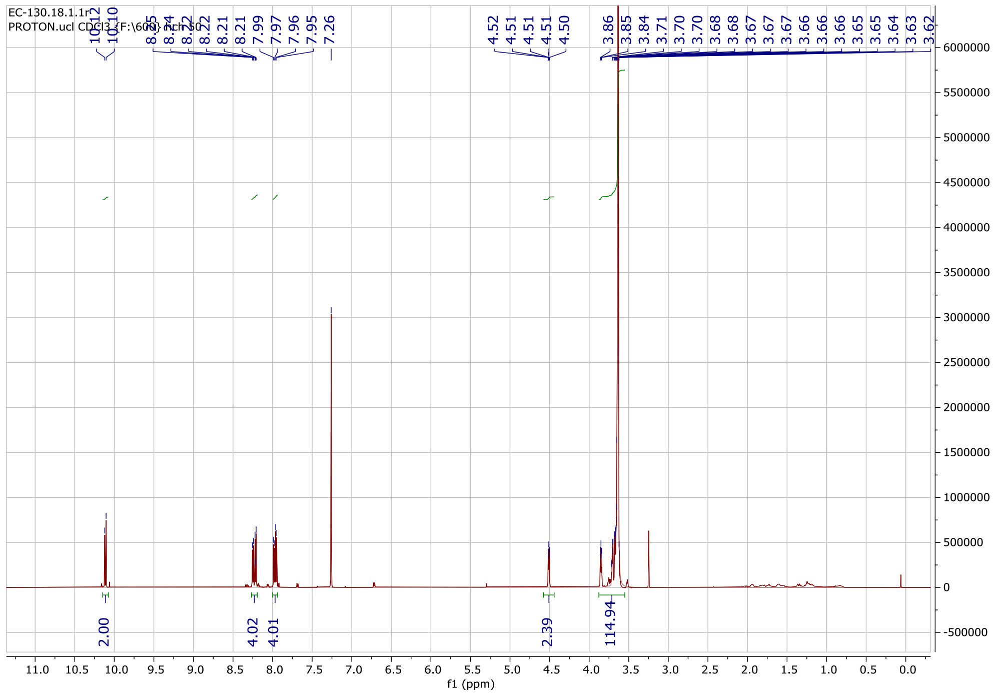

14

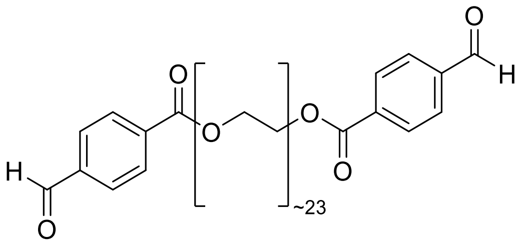

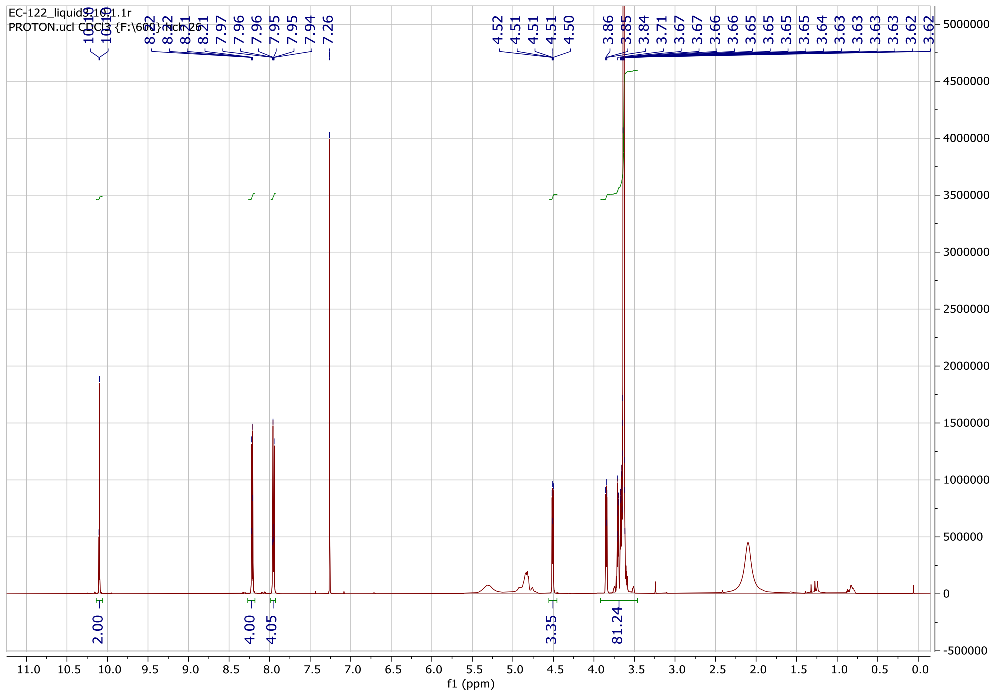

5 References

1 S. Higson, F. Subrizi, T. D. Sheppard and H. C. Hailes, *Green Chem.*, 2016, **18**, 1855–1858.

2 R. J. Petroski, *Synth. Commun.*, 2006, **36**, 1727–1734.

3 S. Higson, University College London, 2017.

4 A. Ros, B. Estepa, R. López-Rodríguez, E. Álvarez, R. Fernández and J. M. Lassaletta, *Angew. Chemie - Int. Ed.*, 2011, **50**, 11724–11728.

5 C. Cao, Y. Shi and A. L. Odom, *Org. Lett.*, 2002, **4**, 2853–2856.

6 S. E. Denmark, J. P. Edwards and O. Nicaise, *J. Org. Chem.*, 1993, **58**, 569–578.

7 S. E. Denmark, J. P. Edwards, T. Weber and D. W. Piotrowski, *Tetrahedron: Asymmetry*, 2010, **21**, 1278–1302.

8 S. C. Cosgrove, J. M. C. Plane and S. P. Marsden, *Chem. Sci.*, 2018, **9**, 6647–6652.

9 M. M.Bradford, *Anal. Biochem.*, 1976, **72**, 248–254.

10 U. Kaulmann, K. Smithies, M. E. B. Smith, H. C. Hailes and J. M. Ward, *Enzyme Microb. Technol.*, 2007, **41**, 628–637.

11 M. F. Villegas-Torres, R. J. Martinez-Torres, A. Cázares-Körner, H. Hailes, F. Baganz and J. Ward, *Enzyme Microb. Technol.*, 2015, **81**, 23–30.

12 M. Höhne, S. Schätzle, H. Jochens, K. Robins and U. T. Bornscheuer, *Nat. Chem. Biol.*, 2010, **6**, 807–813.

13 C. K. Savile, J. M. Janey, E. C. Mundorff, J. C. Moore, S. Tam, W. R. Jarvis, J. C. Colbeck, A. Krebber, F. J. Fleitz, J. Brands, P. N. Devine, G. W. Huisman and G. J. Hughes, *Science (80-.).*, 2010, **329**, 305–310.

14 L. Leipold, D. Dobrijevic, J. W. E. Jeffries, M. Bawn, T. S. Moody, J. M. Ward and H. C. Hailes, *Green Chem.*, 2019, **21**, 75–86.

15 M. Senyel, A. Ünal and Ö. Alver, *Comptes Rendus Chim.*, 2009, **12**, 808–815.

16 D. Enders and H. Schubert, *Angew. Chemie - Int. Ed.*, 1984, **23**, 365–366.
